# Evaluation and comparison of antibiotic susceptibility profiles of *Streptomyces* spp. from clinical specimens revealed common and region-dependent resistance patterns

**DOI:** 10.1038/s41598-022-13094-4

**Published:** 2022-06-07

**Authors:** Lucie Kotrbová, Ana Catalina Lara, Erika Corretto, Josef Scharfen, Vít Ulmann, Kateřina Petříčková, Alica Chroňáková

**Affiliations:** 1 Institute of Soil Biology, Biology Centre Academy of Sciences of the Czech Republic, Na Sádkách 7, 370 05 České Budějovice, Czechia; 2 National Laboratory for Pathogenic Actinomycetes, Trutnov Regional Hospital, Gorkého 77, 541 01 Trutnov, Czechia; 3 Public Health Institute Ostrava, Partyzánské náměstí 2633/7, 702 00, Ostrava, Czechia; 4 Institute of Immunology and Microbiology, 1St Faculty of Medicine, Charles University, Studničkova 7, 128 00 Prague, Czechia

**Keywords:** Microbiology techniques, Antimicrobial therapy

## Abstract

Notwithstanding the fact that streptomycetes are overlooked in clinical laboratories, studies describing their occurrence in disease and potential pathogenicity are emerging. Information on their species diversity in clinical specimens, aetiology and appropriate therapeutic treatment is scarce. We identified and evaluated the antibiotic susceptibility profile of 84 *Streptomyces* clinical isolates from the Czech Republic. In the absence of appropriate disk diffusion (DD) breakpoints for antibiotic susceptibility testing (AST) of *Streptomyces* spp., we determined DD breakpoints by correlation with the broth microdilution method and by the distribution of zone diameters among isolates. Correlation accuracy was high for 9 antibiotics, leading to the establishment of the most valid DD breakpoints for *Streptomyces* antibiotic susceptibility evaluation so far. Clinical strains belonged to 17 different phylotypes dominated by a cluster of strains sharing the same percentage of 16S rRNA gene sequence identity with more than one species (*S. albidoflavus* group*, S. hydrogenans, S. resistomycificus, S. griseochromogenes*; 70% of isolates). AST results showed that *Streptomyces* exhibited intrinsic resistance to penicillin, general susceptibility to amikacin, gentamycin, vancomycin and linezolid, and high percentage of susceptibility to tetracyclines and clarithromycin. For the remaining antibiotics, AST showed inter- and intra-species variations when compared to available literature (erythromycin, trimethoprim-sulfamethoxazole), indicating a region-dependent rather than species-specific patterns.

## Introduction

Despite the great contribution of *Streptomyces* to medicine as a source of many bioactive compounds (antibiotics, immunosuppressants, antifungals and antivirals), they also have a potentially dark side and can cause infections in humans. The most common infection caused by *Streptomyces* is actinomycetoma, an endemic disease associated with *S*. *somaliensis*, *S*. *sudanensis*, *S*. *griseus* and *S. viridis*^1–5^. In addition to mycetoma, *Streptomyces* can also cause rare invasive infections such as pulmonary infections, bacteremias, brain abscesses, peritonitis and other diseases, mainly in immunocompromised patients (reviewed in^6,7^). New approaches using next generation sequencing have recently shown that *Streptomyces* are likely a common part of the human^8–11^. However, the role of clinical *Streptomyces* isolates of unknown aetiology reported in numerous studies^12–14^ remains unclear. Indeed, many *Streptomyces* secondary metabolites exhibit harsh effects in vitro (strong cytotoxic and β-hemolytic activities) and can play a significant role in the human immune response^6^. Moreover, examination of the genome of *Streptomyces* sp. TR1341 isolated from the sputum of an elderly patient with a long history of respiratory problems revealed the presence of several genes for opportunistic colonization of human tissues and for the resistance to antibiotics^15^. Their significance as opportunistic pathogens, the presence and description of possible virulence factors, and antibiotic susceptibility in the context of antibiotic resistance determinant transmission remain to be investigated.

The tendency to isolate streptomycetes from clinical specimens has increased in the last decade^1,6,7,14,16–19^. This may be due to increasing air pollution in industrial and agricultural areas (dust as a vector for *Streptomyces* spores), but also to a growing number of immunodeficient patients caused by extrinsic/external factors (e. g. HIV infection, immunosuppressive drugs, environmental toxins or malnutrition)^20^. Nevertheless, streptomycetes are still neglected in clinical practice, due to a lack of awareness of their clinical relevance and probably due to difficulties associated with their growth characteristics, which complicates their isolation in clinical laboratories. *Streptomyces* are usually slow-growing bacteria that are rapidly overgrown by common respiratory microbiota during routine cultivations. To eliminate the fast-growing bacteria, some mycobacteriological laboratories decontaminate clinical specimens with N-Acetyl-L-Cysteine-Sodium Hydroxide (NALC-NaOH). However, the negative effects of NALC-NaOH on the recovery of mycobacteria and *Nocardia* spp. have been reported^21–23^. Reduction of *Streptomyces* population and species selection cannot be ruled out. Even if *Streptomyces* survive this crude decontamination step, they will most likely be considered as contaminants, since microscopic identification requires an experienced person^24^.

Because of the limited number of studies reporting antibiotic susceptibility profiles of clinical *Streptomyces*, with respect to the total of 674 *Streptomyces* species names validly published^25^, antibiotic therapy against such infections is at best questionable. There is a consistent agreement that clinical *Streptomyces* isolates are susceptible to amikacin, gentamycin, vancomycin, and linezolid. In contrast, the vast majority of clinical isolates tested to date have been resistant to penicillin, the oldest known antibiotic. For the remaining antibiotics, there are differences in susceptibility patterns between species^3,5,13,14,16,26–34^. The results available in the literature suggest species-specific susceptibility, but the number of species-identified clinical isolates is too small to draw a clear conclusion.

The key to predicting the clinical success or failure of a therapy is the antibiotic susceptibility test (AST), i.e., the in vitro response of the pathogen to selected antibiotics. The interpretive criteria to categorize an organism as susceptible (S), intermediate (I) or resistant (R)^35^ are clinical breakpoints. In addition to clinical breakpoints that guide therapy, the concept of epidemiological cut-off values (ECOFF) has been also defined. The ECOFF value serves as threshold for detecting bacteria with resistance mechanisms and for monitoring the evolution of resistance among isolates of the same bacterial species. This value divides a given bacterial population into a wild type group and a group with acquired or mutational resistance to a given drug^36^.

The golden standard in AST is the broth microdilution method (BM). As a fast alternative with a lower cost serves disk diffusion method (DD). Since *Streptomyces* spp. are better adapted to growth on solid/semi-solid media^37^, DD method is more suitable for *Streptomyces* AST. While the DD method has been reported mainly in the characterization of environmental *Streptomyces*^38–40^, its use in clinical setting is not excluded^19^. However, given the lack of breakpoints for *Streptomyces* AST, the results of studies using DD method could be difficult to interpret or even misleading, increasing the likelihood of treatment failure.

The objectives of the present study were: i) to perform a taxonomic classification based on the 16S rRNA gene of 84 clinical isolates of *Streptomyces* collected in Czech Republic, ii) to determine their antibiotic susceptibility profile using the DD method, iii) to evaluate the presence of acquired resistance mechanisms; and iv) to review antibiograms of clinical *Streptomyces* strains available in literature to discuss the suitability of the antibiotic therapy. Since the clinical breakpoints for the evaluation of the DD method results in *Streptomyces* or related organisms are not defined by an official scientific body, we attempted, for the first time, to correlate the DD results with the results of the broth microdilution (BM) techniques standardized by the Clinical and Laboratory Standard Institute (CLSI) for *Streptomyces* related organisms and to set appropriate DD breakpoints for antibiotic profiling of *Streptomyces* spp.

## Materials and methods

### Bacterial isolates acquisition and identification

The study included 84 non-duplicated human clinical isolates collected between 2009 and 2018 at the Trutnov Regional Hospital (29 strains, strain coding TR), the Ostrava Public Healthcare Institute (53 strains, strain coding OS), and the Příbram Regional Hospital (2 strains, strain coding PR). All strains were isolated during routine diagnostics in mycobacteriology laboratories. Strains have been deposited in the Collection of Actinomycetes of the Biology Centre Collection of Organisms (BCCO, www.actinomycetes.bcco.cz). DNA extraction and identification of all isolates was performed according to^41^. The 16S rRNA gene sequences were compared against the type strains database using the Basic Local Alignment Search Tool^42^. The phylogenetic tree was constructed using Geneious (v 8.1.6, http://www.geneious.com, neighbour joining, Tamura-Nei genetic distance model, 1000 replicates). The strains were assigned to different clades according to^43^. The list of all strains and the reference strains most closely related according to 16S rRNA gene similarity and their classification into clusters are reported in Table 1. Strain TR1341 (here assigned to *S. murinus* based on nucleotide similarity of the 16S rRNA gene) was discussed in the previous study^6^. The nucleotide sequences of the 16S rRNA genes of the isolates were deposited in GenBank under the accession numbers MZ393577-MZ393782 (Supplementary Table S1).

**Table 1:** List of clinical and soil isolates used in this study. The closest relative species according to 16S rRNA gene similarity referred as phylotype in this study, the clade assignment, number of strains included in the analysis for each category (Correlation of Minimum Inhibitory Concentrations (MIC) and Disk Diffusion (DD) zone diameter, Antibiotic Susceptibility Testing (AST)), and cluster assignment based on phylogeny analysis conducted in this study (Fig. 1). ND – not defined; * Labeda, D. P. et al. Phylogenetic study of the species within the family *Streptomycetaceae*. *Antonie van Leeuwenhoek* **101**, 73–104 (2012).

Strain ID	The closest relative species according to 16S rRNA gene similarity (phylotype)	Clade assignment*	No. of strains in analysis				Cluster assignment of clinical isolates based on phylogeny analysis
			Correlation MIC – ZD zone diameter			AST	
			Clinical	Soil	Type strain	Clinical	
OS1529, OS2055, OS2660, OS8001, BCCO 10_1711	*S. carpaticus*	Clade 128	2	1	–	4	A (*S. carpaticus*)
OS1126A	*S. ginkgonis*	–	–	–	–	1	
OS967, BCCO 10_1710	*S. xiamenensis*	Clade 128	1	1	–	1	
OS1127, OS282, OS1126 B	*S. albus*	Clade 126	2	–	–	3	B (*S. albus*)
OS17, OS18, OS20, OS21, OS32, 0S33, OS534, OS2864, OS2886A, OS3863, OS3889, OS4303, OS534, OS5590, OS5966, OS6152, OS6180, OS6215, OS6618, OS6629, OS6643, OS6672, OS6764, OS6783, OS6829, OS7188, OS7560, OS8079B, OS8305, OS8560, OS8619, OS8917, TR950, TR979, TR1008, TR1011, TR1048, TR1060, TR1135, TR1206, TR1247, TR1250, TR1349, TR1353, PR198, BCCO 10_0478, BCCO 10_0258, OS19, 0S22, 0S23, OS542, OS2243, OS3864, OS7748, OS8079A, OS10141, TR1041, TR1056, TR1059, TR1117, TR1134, TR1301, BCCO 10_1286, BCCO 10_0550	*S. albidoflavus* group type strains*/S. hydrogenans/S. resistomycificus/S. griseochromogenes*	Clade 112	13	4	–	59	C (*S. albidoflavus*)
TR1355, OS7207, TR1340, TR1147, TR1052	*S. gougerotii/S. rutgersensis/S. diastaticus*	Clade 113	3	–	–	5	D (*S. gougerotii*)
OS6745	*S. intermedius/S. gougerotii/S. rutgersensis/S. diastaticus*	Clade 113	–	–	–	1	
TR1144	*S. thermoviolaceus* subsp. *apingens*	–	1	–	–	1	E (*S. thermoviolaceus*)
TR978	*S. drozdowiczii*	Branch with clade 27	1	–	–	1	F (*S. drozdowiczii*)
TR1341	*S. phaeogriseichromatogenes/S. murinus/S. griseofuscus*	Clade 12	1	–	–	1	G (*S. murinus*)
TR1292, BCCO 10_1719	*S. flavogriseus/S. flavovirens*	Clade 37	1	1	–	1	H (*S. flavogriseus*)
TR1318	*S. flavofuscus/S. baarnensis/S. fimicarius/S. caviscabies*	Branch with clade 35–37	1	–	–	1	
PR181	*S. seoulensis*	–	1	–	–	1	I (*S. seoulensis*)
OS587	*S. xylanilyticus*	–	–	–	–	1	J (*S. xylanilyticus*)
OS4451	*S. thermocarboxydus*	Clade 109	1	–	–	1	K (*S. thermocarboxydus*)
TR1049	*S. variabilis/S. griseoincarnatus/S. erythrogriseus*	Clade 100	1	–	–	1	L (*S. variabilis*)
TR1120	*S. plicatus/S. rochei/S. vinaceusdrappus/S. enissocaesilis*	Clade 119	–	–	–	1	M (*S. plicatus*)
BCCO 10_1622	*S. nanningensis*	–	–	1	–	–	ND
DSM 41685	*S. rameus*	Clade 116	–	–	1	–	ND
BCCO 10_1656	*S. sasae*	–	–	1	–	–	ND
BCCO 10_1683	*S. vinaceus/S. cirratus*	Clade 39	–	1	–	–	ND
BCCO 10_1638	*K. purpeofusca*	Clade 57	–	1	–	–	ND
BCCO 10_0009	*S. arcticus*	Clade 119	–	1	–	–	ND
BCCO 10_1682	*S. wistariopsis*	–	–	1	–	–	ND
BCCO 10_0501	*S. xanthochromogenes/S. mauvecolor/S. michiganensis*	Clade 29	–	1	–	–	ND
BCCO 10_1285	*S. pluricolorescens/S. globisporus/S. rubiginosohelvolus/S. mediolani*	–	–	1	–	–	ND
BCCO 10_1665	*S. rishiriensis*	Branch with clade 21	–	1	–	–	ND
BCCO 10_1630	*S. acidiscabies*	Clade 5	–	1	–	–	ND
BCCO 10_1602	*S. noursei/S. albulus/S. yunnanensis*	Clade 67	–	1	–	–	ND
DSM 40783	*S. coelicolor* A3(2) = *S. violaceoruber*	Clade 103	–	–	1	–	ND

### Antibiotic susceptibility testing

#### Antimicrobials

The antimicrobials used in the study are listed in Table 2. The abbreviations and disk contents are included.

#### Disk diffusion test

 Strains were grown on ISP3 medium^44^ prior AST at 36 °C (28 °C for soil isolates). The 0.5 McFarland suspension was prepared as described by CLSI in the M24 manual^45^. A total of 200 µL of suspension was spread on Mueller–Hinton agar 90-mm plates (Dulab, Dubné, Czech Republic). Commercial antibiotic disks (Bio-Rad, Hercules, CA, USA) were used. Plates were incubated at 36 °C. Zones were measured after 24 or 48 h, depending on the growth rate of the strain. In the case of trimethoprim-sulfamethoxazole, slight growth within the zone was ignored as recommended in guidelines for broth microdilution method^45^.

#### Clinical breakpoints setting

To develop a criterion for interpreting DD results, we followed three different approaches. (1) To correlate DD with BM results for antibiotics available in a commercial BM kit. The minimum inhibitory concentration (MIC) breakpoints chosen to the derive zone diameter (ZD) breakpoints are reported in Table 2. We selected 29 clinical isolates and the number of strains belonging to a given cluster was selected proportionately. To increase the robustness of the correlation analysis, 18 additional soil *Streptomyces* isolates (also deposited in BCCO) and the type strains *S. rameus* DSM 41685 and *S. violaceoruber* DSM 40783 were included in the study. The soil and type strains were subjected to the same procedures as the clinical isolates, except for the growth temperature, which was set to 28 °C. The soil isolates belonged to the same clusters as the clinical ones when possible. A total of 49 *Streptomyces* strains were used in the correlation analysis (Table 1). (2) For antibiotics not available in a commercial BM kit, we proposed at least tentative breakpoints based on the distribution of the ZD data and the class of the antibiotic. (3) For antibiotics not available in a commercial BM kit and without a clear cut-off in the distribution of ZDs, we used arbitrary ZD breakpoints.

#### Minimum inhibitory concentration (MIC) determinations

The BM method for aerobic *Actinomycetes* described by CLSI in the M24 manual^45^ was performed according to the guidelines. The commercial BM kits MIKROLATEST MIC® (Erba Lachema, Brno, Czech Republic) with dried antibiotics were used to determine MICs. Results were read after 72 h (optimal growth of all strains). The strains *Enterococcus faecalis* DSM 2570 and *Escherichia coli* DSM 1103 were used as quality control as recommended in BM kits guidelines.

#### Data analysis

Binary logarithms of the MIC values (mg/L) and average ZDs (mm) were calculated. Correlation between log MIC and average ZDs was tested using the Pearson correlation coefficient. The accuracy limit was set at r > 0.75. Off-scale values were excluded. ZD interpretive susceptibility criteria were derived from the scattergrams of MIC endpoints and the ZD values followed by the error rate-bounded method^46^. The discrepancy percentage between the correlated methods was calculated as very major error (VM; false-susceptible by disk diffusion), major error (M; false-resistant by disk diffusion) and minor error (m; one of the test results is intermediate and the other is susceptible or resistant). Epidemiological cut-off values (CO_WT_) to distinguish wild type and non-wild type populations (group with acquired or mutational resistance to that drug) were determined by automatic calculation for the zone distributions using the Normalized Resistance Interpretation^47^ method for most the abundant species only. The obtained frequencies of resistant phenotypes were compared with those reported in a recent study^14^ of clinical isolates from Spain. The concordance between resistance profiles for overlapping antibiotics was evaluated by Pearson´s chi-squared test (α = 0.01). Only antibiotics with available MIC-DD correlation were included in the comparison (CIP, ERY and SXT). The frequency of resistance to the tested antibiotics, except those with arbitrary breakpoints (SMN, RIF and OFX), was calculated to determine multidrug resistance patterns of the clinical isolates.

#### Review on available antibiograms

Since the studies from clinical disciplines are presumed to use standardized methods for AST evaluation, we focused only on antibiograms reported for clinical *Streptomyces*. If MIC or ZD values were stated in studies^3,12,19,32,48^, we evaluated the susceptibility profile using the breakpoints cited or derived in our study.

For more details about Material and Methods see Supplementary Text S1.

**Table 2:** Results of the correlation analysis between the standard broth microdilution method and the zone diameter distribution, and the results of AST of clinical isolates performed with the disk diffusion method.

	Antimicrobials	Abbreviation	Disk content	Zone diameter breakpoints (mm)			MIC breakpoints (mg/L)			Correlation parameters				% clinical isolates (*n* = *84*)			CO_WT_ of cluster C ≥ (mm)
										Pearson´s r	No. of errors (%)						
				R ≤	I	S ≥	R	I	S		VM	M	m	R	I	S	
Zone diameter breakpoints corelated with MICs	Ampicillin	AMP	10 µg	21	22–24	25	≥ 16	–	≤ 8^A^	−0.96	0	0	5(20)	82	6	12	11
	Amikacin	AKN	30 µg	–	–	30^①^	≥ 16^B^	–	≤ 8^B^	–	–	–	–	–	–	100	28^③^
	Chloramphenicol	CMP	30 µg	23	24–26	27	> 8^C^	–	≤ 8^C^	−0.9	2(7)	0	1(4)	13	2	85	24
	Ciprofloxacin	CIP	5 µg	24	25–29	30	≥ 4^B^	2^B^	≤ 1^B^	−0.89	0	0	9(21)	6	46	48	26
	Erythromycin	ERY	15 µg	22	23–28	29	≥ 8^B^	4^B^	≤ 2^B^	−0.94	0	1(5)	6(29)	87	7	6	9
	Gentamycin	GEN	10 µg	–	–	28^①^	≥ 16^A, D^	8^D, F^	≤ 4^A, D^	–	–	–	–	–	–	100	29
	Penicillin	PEN	10UI	44	45–49	50	> 0.1252^E^	–	≤ 0.12^E^	−0.75	–	–	–	100	–	–	–
	Tetracycline	TET	30 µg	27	28–31	32	> 2^E^	–	≤ 2^E^	−0.93	0	0	2(29)	8	1	91	34
	Trimethoprim-sulfamethoxazole	SXT	1.25 µg + 23.75 µg	14	–	15	≥ 4/76^B^	–	≤ 2/38^B^	−0.87	0	1(9)	–	79^②^	–	21^②^	–
	Vancomycin	VAN	30 µg	–	–	24^①^	–	–	≤ 2^D^	–	–	–	–	–	–	100	25
Tentative breakpoints according ZDs distribution	Amoxicillin	AMX	25 µg	27	28–30	31	–	–	–	–	–	–	–	81	6	13	18
	Amoxicillin-clavulanic acid	AMC	20 µg + 10 µg	20	21–22	23	–	–	–	–	–	–	–	7	6	87	20
	Cefazolin	CZN	30 µg	23	24–29	30	–	–	–	–	–	–	–	98	0	2	12
	Ceftriaxone	CRO	30 µg	23	24–29	30	–	–	–	–	–	–	–	88	5	7	10
	Clarithromycin	CLR	15 µg	21	22–25	26	–	–	–	–	–	–	–	7	4	89	22
	Doxycycline	DOX	30 µg	27	28–31	32	–	–	–	–	–	–	–	7	1	92	35
	Minocycline	MNO	30 µg	27	28–31	32	–	–	–	–	–	–	–	4	2	94	36
Arbitrary breakpoints	Rifampicin	RIF	5 µg	20	21–29	30	–	–	–	–	–	–	–	11	27	62	24^③^
	Streptomycin	SMN	10 µg	20	21–29	30	–	–	–	–	–	–	–	54	39	7	11
	Ofloxacin	OFX	5 µg	20	21–29	30	–	–	–	–	–	–	–	17	76	7	18
Only BM method performed	Linezolid	LIZ	–	–	–	–	–	–	≤ 8^B^	–	–	–	–	–	–	100^④^	–
	Ampicillin-sulbactam	AMS	–	–	–	–	≥ 16	–	≤ 8^A^	–	–	–	–	48^④^	–	52^④^	–

List of antibiotics, their abbreviations, and disk concentration used in the study are provided. Antibiotics are grouped into categories according to zone diameter breakpoint settings. Table shows the proposed zone diameter breakpoints based on ZD-MIC correlation analysis altogether with ZDs distribution among clinical strains, proposed MIC breakpoints according to available breakpoints values in CLSI and EUCAST guidelines and according to ZDs distribution among clinical strains. Parameters of the correlation analysis are provided: Pearson correlation coefficient (r), VM – very major error, false-susceptible by DD; M – major error, false-resistant by DD; m- minor error, one of the test results is intermediate and the other is susceptible or resistant. Antibiotic susceptibility distribution of clinical strains based on proposed ZD breakpoints and wild type cut-off (CO_WT_) calculated for clinical strains in the cluster C. Abbreviations: R = resistant, I = intermediate susceptible, S = susceptible; ① minimum ZD values were proposed as susceptibility breakpoints (type strain DSM 40783 was excluded for vancomycin and BCCO 10_1638 for amikacin), since predominantly susceptible strains (BM method) were present; ② only 78 clinical isolates were included, cluster D was excluded; ③ abnormal standard deviation; ④ only 29 clinical isolates were tested. References: A—Larruskain, J., Idigoras, P., Marimón, J. M., Pérez-Trallero, E. Susceptibility of 186 *Nocardia* sp. isolates to 20 antimicrobial agents.* Antimicrob. Agents. Chemother.* **55**, 2995–8 (2011); B—CLSI. Susceptibility testing of mycobacteria, and other aerobic actinomycetes. 2nd Ed. *CLSI Guideline M24* (2011); C—Dragomirescu, C. C. *et al.* Antimicrobial susceptibility testing for *Corynebacterium* species isolated from clinical samples in Romania. *Antibiotics* **9**, 1–9 (2020); D—CLSI. Methods for antimicrobial dilution and disk susceptibility testing of infrequently isolated or fastidious bacteria. 3rd Ed. *CLSI Guideline M45* (2015); E—EUCAST. Testing Breakpoint tables for interpretation of MICs and zone diameters. Available from: https://www.eucast.org/fileadmin/src/media/PDFs/EUCAST_files/Breakpoint_tables/v_10.0_Breakpoint_Tables.pdf (2020).

## Results

### Strain taxonomy

**Figure 1:**
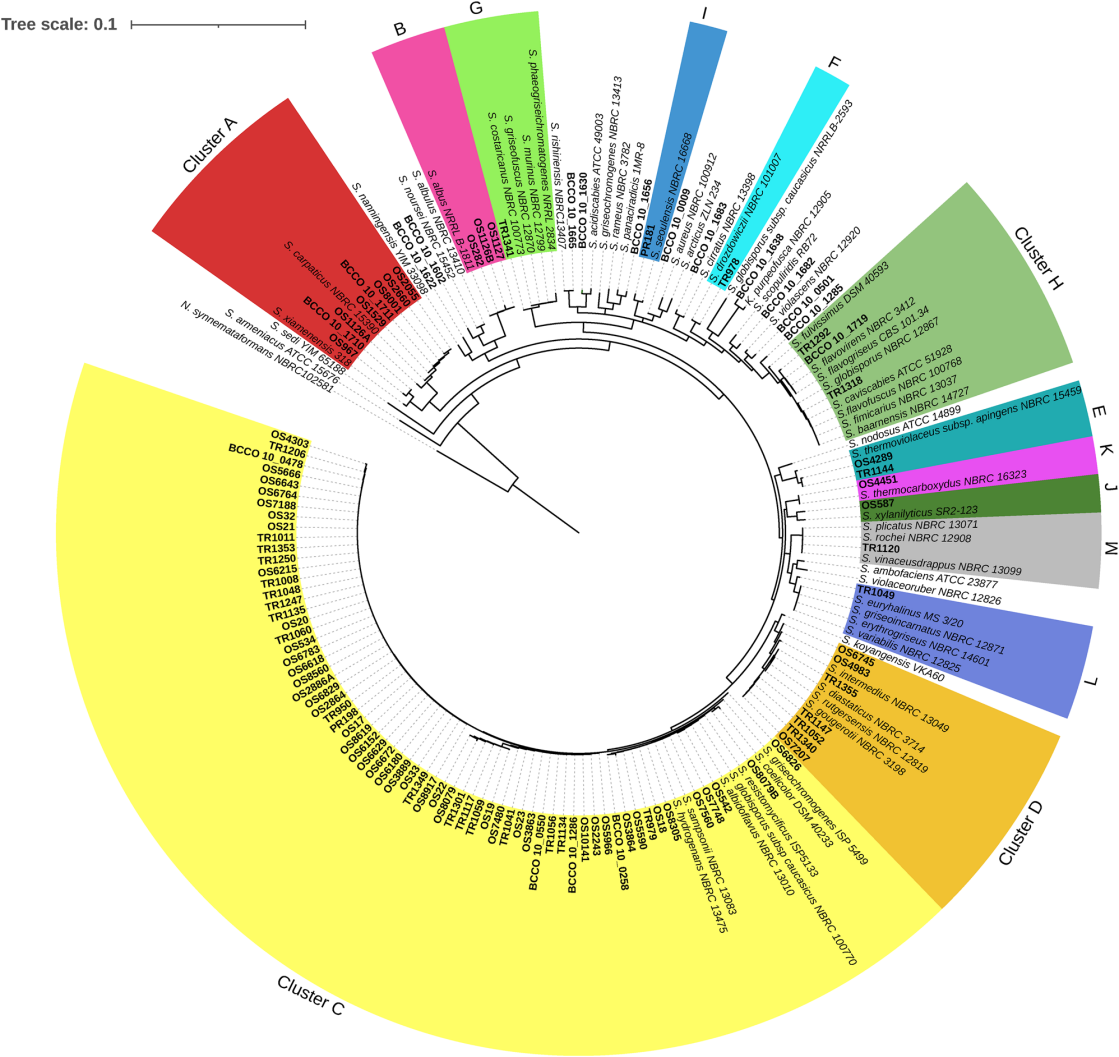
Neighbor-Joining tree of *Streptomyces* strains used in this study based on the 16S rRNA gene. Clinical isolates are highlighted in bold. Individual phylogenetic clusters are distinguished by colour and assigned letter codes. The 16S rRNA gene sequence of *Nocardioides synnemataformans* NBRC102581 served as the outgroup.

The selected 84 clinical *Streptomyces* strains belong to 16 different phylotypes (Table 1). Most strains (83%) have the same percentage of sequence identity (PID) with more than one *Streptomyces* species due to insufficient variation in their 16S rRNA gene sequence (all clinical strains PID > 99%, except OS587 with PID 98.88%). The phylogenetic tree of the clinical *Streptomyces* strains was complemented with environmental and type strains (Fig. 1). The clinical strains were then assigned to 13 phylogenetic clusters, with cluster C (*S. albidoflavus* group/*S. hydrogenans*/*S. resistomycificus*/*S. griseochromogenes*) comprising 70% of all clinical isolates.

### ZD breakpoints setting

To derive ZD interpretive criterion, 3 different approaches were followed: i) Correlation of BM MICs values with zone diameters when antibiotics available in commercial BM kits; ii) Tentative breakpoints setting based on the distribution of ZD data and class of the antibiotic for antibiotics without correlation analysis; iii) Arbitrary ZD breakpoints when the distribution of ZDs did not reveal a clear cut-off for S-R category for antibiotics without correlation analysis.

#### Correlation analysis-based breakpoints

**Figure 2:**
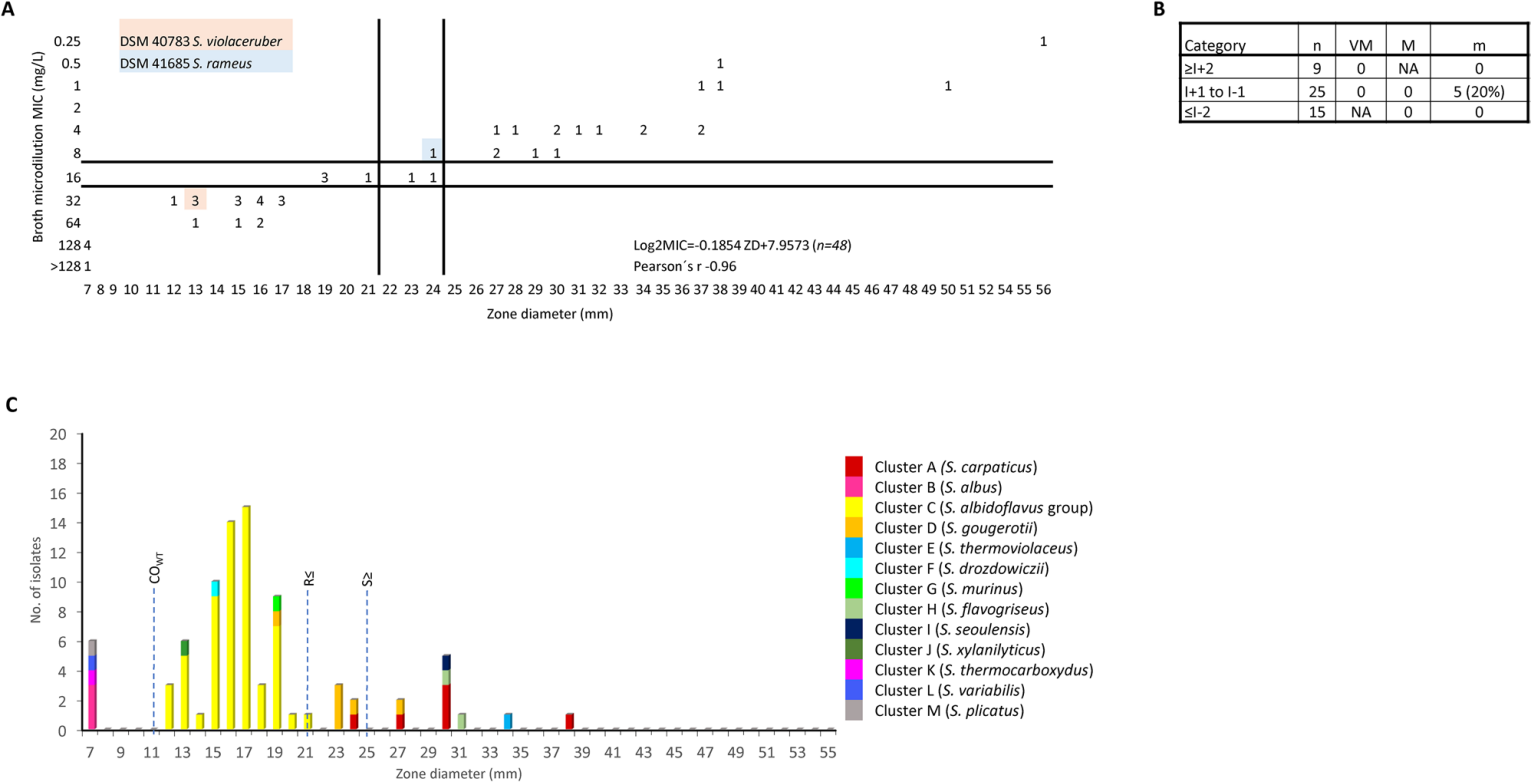
Ampicillin. Results of the correlation analysis of BM and DD methods followed by susceptibility testing of clinical isolates. (**A**) Scattergram comparing the results of broth microdilution MICs (mg/L) and zone diameters (mm) for 49 *Streptomyces* strains. The lines represent the proposed ZD interpretive criteria. (**B**) The table displays the number of tested isolates (n), very major error (VM), major error (M) and minor error (m). (**C**) The graph depicts the zone diameters distribution for 84 clinical *Streptomyces* strains. Dotted lines represent proposed zone diameter breakpoints (R—resistant category, S—susceptible category) and the CO_WT_ value. Individual phylogenetic clusters are distinguished by colour. Based on the scattergram data, distribution of MICs and zone diameters, breakpoints were set as R ≤ 18 mm, I = 19–24 mm and S ≥ 25 mm, with no very major or major error and 4% of minor error (1 strain). However, when evaluating the zone size distribution of clinical strains, the CO_WT_ of dominant cluster C was calculated to be 11 mm, indicating that the entire cluster C is resistant to ampicillin. Therefore, to minimise the risk of setting clinical breakpoints which split the resistant population (dominant cluster C) and in order to prevent misclassification of resistant strains as intermediate (I), we propose to use interpretive breakpoints as R ≤ 21 mm, I = 22–24 mm, and S ≥ 25 mm.

We found a strong negative correlation (Pearson´s correlation r ˂ −0.89) between MIC and ZD values for AMP, ERY, TET, CMP, CIP, and PEN (−0.96, −0.94, −0.93, −0.90, −0.89 and −0.75, respectively). The proposed ZD breakpoints and the CO_WT_ values are shown in Table 2. Discrepancy percentages of proposed breakpoints were in acceptable ranges. Scattergrams and discrepancy percentages are shown in Fig. 2 and Supplementary Figures S1–S7. Evaluation of the correlation between MIC and ZD values for SXT was problematic due to difficulties in determining the MIC and ZD endpoints, which is likely reflected in a weak correlation (Pearson´s r = −0.60, *n* = 36, data not shown). In addition, strong disagreement between categories occurred in 27% of strains. This was partially resolved by prolonging the incubation in the DD method to 48 h. However, unsolved inconsistency persisted for cluster D strains (r = −0.87 when cluster D strains were excluded). Incomplete interpretive breakpoints were proposed for VAN, GEN, and AKN, since only susceptible populations were available. Therefore, as ZD susceptibility breakpoints were chosen the minimum ZD values measured while susceptible by BM method.

#### Breakpoints based on ZD data distribution and on antibiotic class

The ZD distribution of CZN, CRO and CLR allowed us to visually define the R-S cut-offs, as most of the tested clinical *Streptomyces* strains were indisputably resistant (ZD 7–20 mm) or susceptible (ZD more than 27 mm). The antibiotic class membership and similarity of MIC breakpoint values for unrelated genera (available in the AST guidelines) were considered for MNO, DOX, AMX and AMC (Supplementary Figures S8 and S9).

#### Arbitrary ZD breakpoints

The ZD distribution of DOX, OFX and SMN did not allow us to visually define the R-S cut-offs, and with the lack of MIC–ZD correlation analysis, we arbitrarily defined the ZD breakpoint values as R ≤ 20 mm, I = 21–29 mm, S ≥ 30 mm (Supplementary Figure S10A-C).

### Antibiotic susceptibility testing of clinical *Streptomyces* isolates

The percentages of resistant, intermediate susceptible and susceptible strains are shown in Table 2. The CO_WT_ values defining the cut-offs for the wild type and non-wild type population of the strains in cluster C are also shown in Table 2. The resistance patterns of each *Streptomyces* phylotype and the assignment of the corresponding graphs are summarized in Table 3.

**Table 3:** Results of clinical isolates antibiotic susceptibility.

*Streptomyces* phylotype	Cluster assignment based on phylogeny analysis	No. of strains	No. of susceptible (intermediate susceptible) strains in group ^A^										No. of susceptible (intermediate susceptible) strains in group ^B^							No. of susceptible (intermediate susceptible) strains in group ^C^			No. of susceptible (intermediate susceptible) strains in group ^D^		
			AMP	CIP	CMP	ERY	TET	GEN	PEN	SXT	AKN	VAN	AMX	AMC	CZN	CRO	CLR	DOX	MNO	SMN	RIF	OFX	No. of strains	AMS	LIZ
			Fig. 2	Fig. S1	Fig. S2	Fig. S3	Fig. S4	Fig. S7	Fig. S6	Fig. S5	Fig. S7	Fig. S7	Fig. S9	Fig. S9	Fig. S8	Fig. S8	Fig. S8	Fig. S9	Fig. S9	Fig. S10	Fig. S10	Fig. S10			
*S. carpaticus*	A	4	3(1)	4	0	0	4	4	0	4	4	4	4	4	0	4	0(1)	4	4	0	0(1)	4	2	2	2
*S. ginkgonis*	A	1	1	1	0	0	1	1	0	1	1	1	1	1	0	1	0	1	1	0	0	1	0	–	–
*S. xiamenensis*	A	1	1	1	0	0	1	1	0	1	1	1	1	1	0	1	0	1	1	0	0(1)	0(1)	0	–	–
*S. albus*	B	3	0	1(2)	0	0	0	3	0	3	3	3	0	0	0	0	0(2)	0	0(1)	2(1)	0(1)	0	2	0	2
*S. hydrogenans/S. resistomycificus/S. griseochromogenes/S. albidoflavus* group strains	C	59	0	26(32)	57(2)	0(4)	58(1)	59	0	0	59	59	0(1)	56(3)	0	0	59	59	59	0(24)	40(16)	0(55)	13	4	13
*S. gougerotii/S. rutgersensis/S. diastaticus*	D	5	1(4)	4(1)	5	0	5	5	0	ND	5	5	1(4)	5	0	0	5	5	5	0(4)	5	0(5)	3	3	3
*S. intermedius/S. gougerotii/S. rutgersensis/S. diastaticus*	D	1	0	0(1)	1	0	1	1	0	ND	1	1	0	0(1)	0	0	1	1	1	0	1	0(1)	0	–	–
*S. thermoviolaceus*subsp. *apingens*	E	1	1	1	1	1	1	1	0	1	1	1	1	1	1	1	1	1	1	1	1	1	1	1	1
*S. drozdowiczii*	F	1	0	1	1	1	0	1	0	1	1	1	0	1	0	0	1	0	1	0(1)	1	0	1	0	1
*S. phaeogriseichromatogenes/S. murinus/S. griseofuscus*	G	1	0	0	0	1	0	1	0	0	1	1	0	1	0	0	1	0	0	1	0(1)	0	1	1	1
*S. flavogriseus/S. flavovirens*	H	1	1	0(1)	1	0(1)	1	1	0	1	1	1	1	1	0	0(1)	1	1	1	1	1	0(1)	1	1	1
*S. flavofuscus/S. baarnensis/S. fimicarius/S. caviscabies*	H	1	1	1	1	0	1	1	0	1	1	1	1	1	0	0(1)	1	1	1	0	1	0	1	1	1
*S. seoulensis*	I	1	1	0(1)	1	1	1	1	0	1	1	1	1	1	1	0(1)	1	1	1	1	1	0	1	1	1
*S. xylanilyticus*	J	1	0	0(1)	1	0	1	1	0	0	1	1	0	0(1)	0	0	1	1	1	0	1	0(1)	1	1	1
*S. thermocarboxydus*	K	1	0	0	1	0(1)	1	1	0	0	1	1	0	0	0	0	1	0(1)	1	0(1)	0	0	1	0	1
*S. variabilis/S. griseoincarnatus/S. erythrogriseus*	L	1	0	0	1	1	0	1	0	1	1	1	0	0	0	0	1	1	1	0(1)	0	0	1	0	1
*S. plicatus/S. rochei/S. vinaceusdrappus/S. enissocaesilis*	M	1	0	0	0	0	0	1	0	1	1	1	0	0	0	0	1	0	0(1)	0(1)	0	0	0	–	–

Numbers represent the quantity of susceptible and intermediate susceptible (in the brackets) strains. Supplementary figures with details of the AST results are referred for each antibiotic. Abbreviations: ND – not determined. Note: ^A^Breakpoints set by correlation analysis; ^B^Breakpoints based on ZD data distribution and on class of antibiotic; ^C^Arbitrary ZD breakpoints; ^D^Only MIC values for 29 clinical strains; AMP – ampicillin, CIP – ciprofloxacin, CMP – chloramphenicol, ERY – erythromycin, TET – tetracycline, GEN – gentamycin, PEN – penicillin, SXT – trimethoprim-sulfamethoxazole, AKN – amikacin, VAN – vancomycin, AMX – amoxicillin, AMC – amoxicillin-clavulanic acid, CZN – cefazolin, CRO – ceftriaxone, CLR – clarithromycin, DOX – doxycycline, MNO – minocycline, SMN – streptomycin, RIF – rifampicin, OFX – ofloxacin, AMS – ampicillin-sulbactam, LIZ – linezolid.

A high percentage of resistant clinical strains was found in case of penicillin group antibiotics: PEN (100%), AMP (82%) and AMX (81%). The enrichment of AMX with clavulanic acid rapidly decreased the resistance of clinical isolates to 7%. A high resistance frequency was also found in case of CZN, CRO, ERY and SXT: 98%, 88%, 87% and 79%, respectively. In contrast, all clinical isolates were susceptible to AKN, GEN and VAN. A high frequency of susceptible clinical strains was found in case of tetracycline group antibiotics (MNO, DOX, TET), CLR, CMP and CIP: 94–91%, 89%, 85% and 49% (plus 45% in “I” category), respectively. A high frequency of susceptible or intermediate susceptible clinical strains was also found for RIF (62% and 27% as “S” and “I” category) and OFX (7% and 76% as S and I category). However, due to the lack of reliable breakpoints (ZD breakpoints were set arbitrarily) these results need further validation. Within the tested cluster C strains, 3.4%, 3.4%, 6.7%, 8.5%, 1.7% and 1.7% were non-wild type in case of AMX, CZN, CIP, TET, DOX and VAN, respectively. For AKN and RIF, non-wild type populations were not determined, as the cluster C zone diameter datasets have an abnormal distribution. For the remaining tested antibiotics, all strains were wild types.

For AMS and LIZ, we performed only the BM method and only 29 clinical isolates were included. All isolates were susceptible to LIZ. In case of AMS, the MIC values of 83% of tested clinical isolates decreased by 1 or more dilutions compared to the MIC value of AMP alone, i.e., the susceptibility of the strains increased (24% of clinical isolates changed susceptibility category from R to S).

Most isolates were resistant to 7 drugs (53 isolates), with a minimum resistance to 1 drug (TR1144 resistant only to PEN) and with maximum multi-drug resistance to 12 drugs occurring in 2 isolates in cluster B (OS282 and OS1126B) and strain TR1341 of cluster G (Supplementary Figure S11).

**Figure 3:**
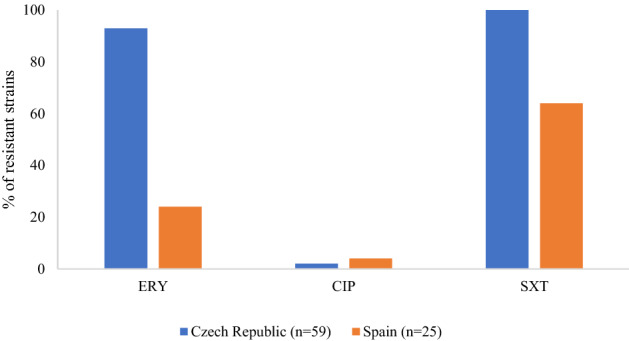
The occurrence of resistant clinical isolates of cluster C in Czech Republic and Spain. Comparison of AST results preformed in this study with the ones reported in a recent study by^14^. ERY - erythromycin, CIP - ciprofloxacin, SXT - trimethoprim-sulfamethoxazole.

The comparison of AST results between clinical isolates of the same cluster (cluster C) collected in Spanish provinces (25 isolates)^14^ and those in our study (59 isolates) is shown on Fig. 3. The proportion of resistant isolates significantly differs by country for ERY (*X*^*2*^ = 42.314; p ˂ 0.01) and SXT (*X*^*2*^ = 23.789; p ˂ 0.01). The frequency of resistant isolates is 93% and 100% in Czech Republic compared to 24% and 64% in Spain, respectively. The proportion of resistant isolates does not differ for CIP (*X*^*2*^ = 0.401; p = 0.818).

### Review on antibiograms

Antibiograms of clinical *Streptomyces* available in the literature are summarized in Supplementary Table S2 (December 2021). We confirmed general susceptibility to AKN, GEN, VAN and LIZ, and resistance to PEN. For the remaining antibiotics, there are inter- and intra-species variations with unexplained drivers.

## Discussion

Although *Streptomyces* is increasingly emerging in clinical settings, there is little information on the aetiology, species distribution and antibiotic susceptibility profiles, with respect to the high number of *Streptomyces* species described to date. These are mostly case reports^5,19,28–30,32–34,49–51^, with only scarce works addressing the large cohorts of clinical isolates^12–14^. Together with these, our work brings a new perspective on the presence and diversity of *Streptomyces* spp. in human microbiome, and points, that streptomycetes are important in clinical samples and should receive greater attention.

Here we present identification and AST of 84 clinical *Streptomyces* isolates collected in Central Europe (Czech Republic). For the first time, we performed a correlation between the BM and DD method in AST of *Streptomyces*. Most of the strains in our study were isolated from patients suffering from chronic respiratory disease (59.5%), although it is not clear whether they were the cause of the disease, represented a secondary bacterial infection, or they were common colonizers of the human body. Although 17 different phylotypes were identified among the clinical *Streptomyces* in our study, only 3 of them have been reported as causative organisms of human diseases before: *S. albus* and *S. thermoviolaceus* in pulmonary infections^27,50,52^, *S. albus* as a causative agent of mycetoma^33^ and *S. thermocarboxydus* in keratitis^53^. A recent study^14^ identified 6 other phylotypes identical to those presented in our study (*S. albidoflavus*, *S. rutgersensis*, *S. rochei*, *S. drozdowiczii*, *S. xylanilyticus* and *S. carpaticus*), but their clinical relevance is unknown. The phylotypes reported in our study as clusters H, I, L, M, and *S. ginkgonis*, with *S. xiamenensis* of cluster A are associated with clinical specimens for the first time. It is interesting to note that cluster C, which is the most abundant in our study (70.2% of clinical isolates), has been reported only in another study so far, and moreover with much less proportion (~ 13% of clinical isolates)^14^. This could be due to regional differences in agriculture, industry and lifestyle, that could play a role in the colonization of humans by different *Streptomyces* species, as well as due to complicated taxonomic issues. Taxonomy of *Streptomyces* species is a complex task involving the identification of genotypic and phenotypic characters^54^. The sequences in the GenBank database are updated literally every minute, and therefore the reports on species identified decades ago are questionable (e. g. 28 *Streptomyces* isolates of *S. griseus*^12^) and thus difficult to compare.

To evaluate the AST of *Streptomyces* spp. we chose the modified Kirby-Bauer method correlated with broth microdilution method (CLSI guidelines for *Nocardia* and other aerobic actinomycetes^45^) for 10 antibiotics. Since establishing clinical breakpoints is a challenging task, all available MIC breakpoints for *Streptomyces* spp. were selected as alternatives based on their taxonomic classification. If official MIC breakpoints for *Streptomyces* spp. are established by an international committee, the DD breakpoints proposed in this study can be easily adjusted using the scattergrams provided.

The correlations were excellent for all tested antibiotics (Pearson´s correlation coefficient ranged from −0.89 to −0.96), except for PEN and SXT. *Streptomyces* species are known for their benzylpenicillin resistance^55^, thus all the strongly resistant values outside the MIC range (> 8 mg/L) had to be excluded from the correlation calculation (28 strains). This might have led to less accurate results. In case of SXT, the inconsistency in reading the MIC endpoint occurred. Due to the uneven growth of streptomycetes in liquid cultures (growth in clumps), it is difficult to detect wells with partial growth (20% lower detection rate comparing to control well as recommended by CLSI) or determine a clear cut-off, while slight growth is neglected. Moreover, the unresolved disagreement between methods occurred in cluster D (*S. gougerotii*). Although SXT is recommended worldwide for the treatment of actinomycetoma^56^ as an empirical antibiotic therapy, we believe that the treatment of *Streptomyces* infections with SXT is probably inappropriate because of the low accuracy of the results as well as the high percentage of resistant strains in our study.

The variability of the species in our study is limited and is mainly represented by cluster C strains (70.2%), which have the same or very similar resistance patterns. Considering the species-specific susceptibility profile of *Streptomyces*^57^, the notably high percentages of resistance to some antibiotics (AMP, SXT and ERY) may be biased by the large proportion of one phylotype in our data set. Therefore, we compared AST profiles from our study with those for clinical species previously published (Supplementary Table S2). Our data confirmed a general susceptibility of *Streptomyces* to AKN, GEN, VAN and LIZ. The only discrepancy found in the literature is a strain of *S. griseus* resistant to AKN with MIC of 16 mg/L^12^ described as susceptible in the study, but resistant according to current guidelines^45^. However, the result is questionable, since the AST procedures as well as the methods for identification have changed since the study was published (1990). These antibiotics are associated with the treatment of complicated multi-drug resistant infections and are often reserved as drugs of last resort, some of which have significant side effects^58–62^. On the contrary, there is intrinsic resistance to PEN, usually recommended as first-line therapy for the treatment of respiratory diseases and pneumonia^63^. For the remaining antibiotics, there is a considerable variability in the susceptibility profiles of *Streptomyces* spp., for some of them with high percentage of resistance (cephalosporins, CIP, ERY) or susceptibility (CLR, tetracyclines, imipenem). Thus, if antibiotic other than the safe one must be used, AST of the causative organism is recommended as well as species identification.

The most interesting property of *Streptomyces* is their ability to produce antibiotics. Since antibiotic resistance genes are thought to originate from antibiotic-producing bacteria, the likelihood of multi-drug resistance (MDR) occurrence is high. For example, there are more than 100 drug resistance gene homologues in the chromosome of *Streptomyces coelicolor* A3(2)^64^ and the presence of the Van cluster (*vanSRJKHAX*) associated with inducible resistance to vancomycin has been reported, too^65^. Nevertheless, there are indications that most of the MDR systems are suppressed under laboratory conditions and *Streptomyces* species are therefore generally considered to be drug sensitive^66^. In our study, we found strains with a wide range of resistance patterns, from generally susceptible to MDR strains. It is noteworthy that one of the most resistant clinical strains in our study, *Streptomyces* sp. TR1341, originated from a patient with multiorgan tuberculosis, relapsing respiratory infections and chronic obstructive pulmonary disease with a long-term and repeated antibiotic therapy^6^. This phenotype was supported by genomic analysis, which revealed the presence of 41 known resistance models in its genome^15^.

Even though non-wild type strains were found in clinical isolates of cluster C, there is no obvious shift in susceptibility category within strains of same phylotype. The ZD value of the only one non-wild type isolate of cluster C that changed susceptibility category compared to wild type isolates balances at the ZD breakpoint value. Therefore, our data suggest a rather species-specific susceptibility profile of *Streptomyces.* To confirm our findings, we compared the antibiograms of the cluster C strains with those presented in the only study on clinical isolates of *S. albidoflavus* (Spain) in the literature to date^14^. All our isolates from cluster C were resistant to ERY and SXT, as reflected also in a low CO_WT_ value in case of ERY (CO_WT_ for SXT was not calculated since all strains lacked an inhibition zone). Contrary, only 24% of *S. albidoflavus* isolates from Spain were resistant to ERY. This discrepancy suggests local adaptation of the cluster C species to unique selection pressures in different regions, including differences in agriculture, industry, lifestyle, and also antibiotic and other drug policy (higher consumption of SXT and macrolides in Czech Republic compared to Spain)^67,68^. Spontaneous mutations conferring ERY-resistance under selection pressure have already been demonstrated in the model actinomycetes *S. coelicolor* and *S. lividans*^69^ and are associated with point mutations in *rrnA*-23S rRNA and *rrnC*-23S rRNA^70^. Since SXT resistance determinants are located on mobile elements such as small plasmids and gene cassettes^71^, horizontal gene transfer under appropriate conditions is a possibility, although it has not yet been documented for *Streptomyces*.

In conclusion, we proved a generally high suitability and accuracy of the disk diffusion method for the AST of *Streptomyces* spp. by correlation with the gold-standard microdilution broth method for 9 antibiotics (SXT remains the questionable due to unresolved ambiguity in cluster D). This led to the determination of DD susceptibility breakpoints derived from MIC breakpoints for *Streptomyces*—related organisms. To the best of our knowledge, these are the most valid DD breakpoints for *Streptomyces* reported to date. Tentative breakpoints have been proposed for 10 additional antibiotics, however, these breakpoints were designed primarily for the purpose of this study. Further analyses, such as correlation analyses, are recommended. All tested clinical isolates were susceptible to AKN, GEN, VAN and LIZ which is in agreement with literature, therefore these antibiotics can be chosen as empiric treatment for *Streptomyces*-associated infections. A low percentage of resistant isolates (˂ 10%) was found in our study for tetracyclines, CLR, CIP and AMC, however, data for CIP and AMC susceptibility differs in literature (Supplementary Table 2). In contrast, *Streptomyces* are intrinsically resistant to penicillin, and have a high percentage of resistance to cephalosporins. Other antibiograms (ERY and SXT) appear to be regionally driven, rather than species-specific and thus AST must be performed prior therapy. The treatment with ERY and SXT associates with a high risk of failure due to acquired resistance and should be reconsidered. Our study also emphasizes that *Streptomyces* are emerging in clinical practice, although still largely neglected, and points out the importance of optimizing techniques for selective isolation from clinical specimens. As awareness of streptomycetes infections in humans has increased, it is desirable to continue investigating their virulence factors and clinical relevance.

## Supplementary Information

Supplementary Information 1.

Supplementary Information 2.

Supplementary Information 3.

Supplementary Information 4.

Supplementary Information 5.

Supplementary Information 6.

Supplementary Information 7.

Supplementary Information 8.

Supplementary Information 9.

Supplementary Information 10.

Supplementary Information 11.

Supplementary Information 12.

Supplementary Information 13.

Supplementary Information 14.

Supplementary Information 15.

## Data Availability

The datasets used and/or analysed during the current study available from the corresponding author on reasonable request.
