# Supplementary material for: Evaluation and comparison of antibiotic susceptibility profiles of Streptomyces spp. from clinical specimens revealed common and region-dependent resistance patterns
Source: Sci Rep. 2022 Jun 7;12:9353. doi: 10.1038/s41598-022-13094-4 (PMC9174267; doi:10.1038/s41598-022-13094-4)

Supplementary Figure S1. Ciprofloxacin. Results of correlation analysis of BM and DD methods followed by susceptibility testing of clinical isolates. A) Scattergram comparing the results of broth microdilution MICs (mg/L) and zone diameters (mm) for 49 *Streptomyces* strains. The lines represent the proposed ZD interpretive criteria. **B)** The table display number of isolates tested (n), very major error (VM, major error (M) and minor error (m). **C)** The graph depicts zone diameters distribution for 84 clinical *Streptomyces* strains, doted lines represents proposed zone diameter breakpoints (R - resistant category, S - susceptible category) and CO_{WT} value.

A.

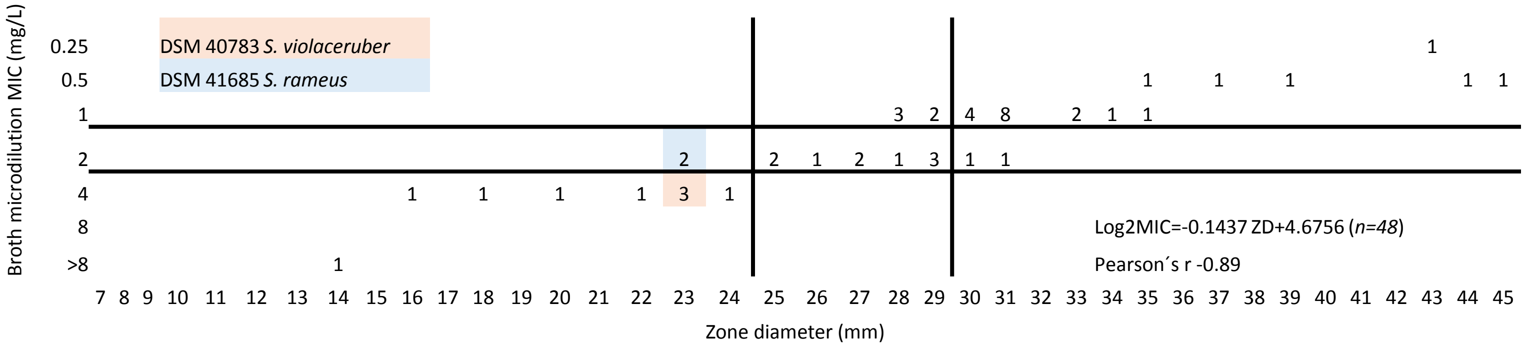

B.

Category	n	VM	M	m
≥I+2	1	0	NA	0
I+1 to I-1	42	0	0	9 (21 %)
≤I-2	6	NA	0	0

C.

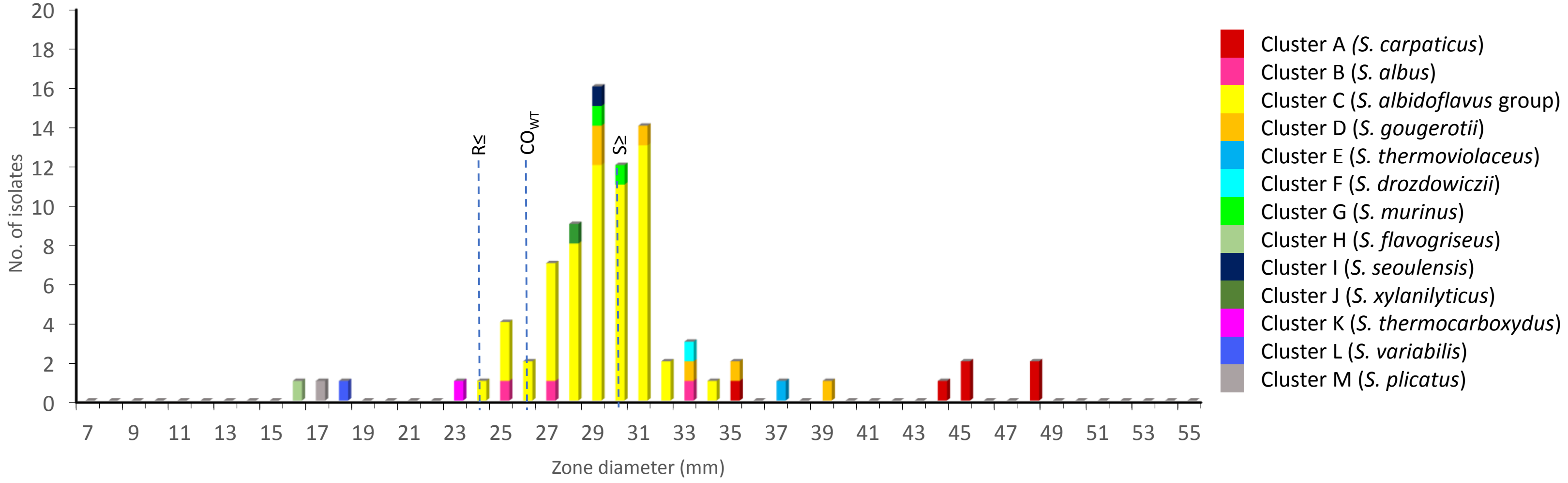

Supplement: Supplementary file 1 — Supplementary Information 1. [file 41598_2022_13094_MOESM1_ESM.pdf]
