# Supplementary material for: Evaluation and comparison of antibiotic susceptibility profiles of Streptomyces spp. from clinical specimens revealed common and region-dependent resistance patterns
Source: Sci Rep. 2022 Jun 7;12:9353. doi: 10.1038/s41598-022-13094-4 (PMC9174267; doi:10.1038/s41598-022-13094-4)

Supplementary Figure S5. Trimethoprim-sulfamethoxazole. Results of correlation analysis of BM and DD methods followed by susceptibility testing of clinical isolates. A) Scattergram comparing the results of broth microdilution MICs (mg/L) and zone diameters (mm; 48 hours of incubation) for 45 *Streptomyces* strains. The lines represent the proposed ZD interpretive criteria. **B)** The table display number of isolates tested (n), very major error (VM, major error (M) and minor error (m). **C)** The graph depicts zone diameters distribution for 84 clinical *Streptomyces* strains, doted lines represent proposed zone diameter breakpoints (R - resistant category, S - susceptible category).

Note: The growth of *Streptomyces* spp. in the broth microdilution wells with decreasing ATB concentration was gradual. There was no clear cut-off and the endpoint could be read in the range of more than one well. Similarly, ambiguity occurred in the growth rate around the antibiotic disk. In addition, 13 of the samples had a MIC endpoint 4/76 mg/L or > 4/76 mg/L („R" category) but zone sizes were measured in the ranges of 23-39 mm indicating rather „*S*" category. After incubating them 24 hours more, all strains grew to disk, i.e. susceptible strains became resistant. However, this trend was not observed in the clinical strains of cluster D. All strains classified as resistant by the BM method had no zone of inhibition after 48 hours.

A.

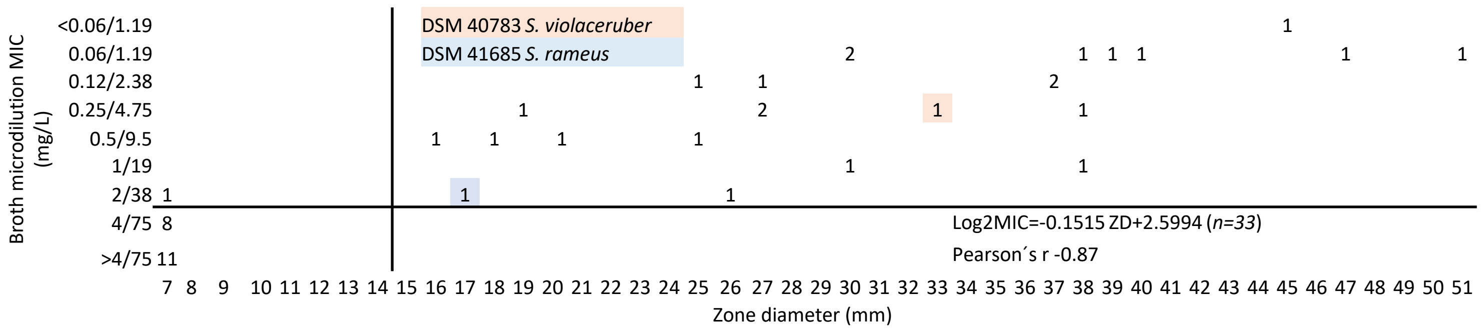

B.

Category	n	VM	M
≥R+1	11	0	NA
R+S	11	0	1 (9 %)
≤S-1	23	NA	0

C.

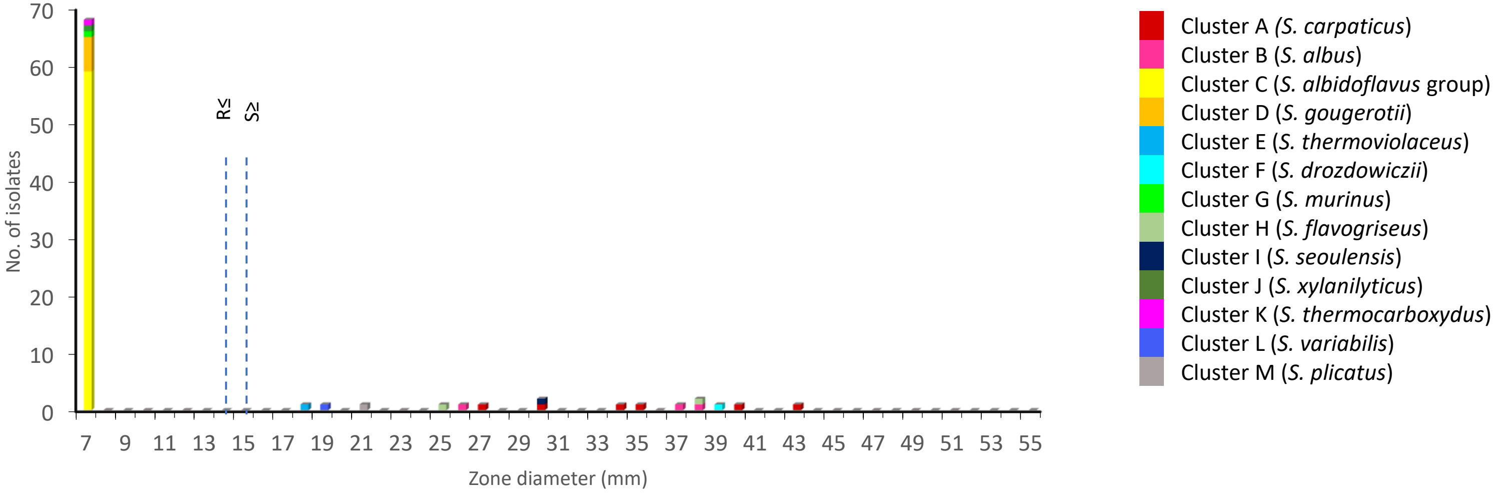

Supplement: Supplementary file 5 — Supplementary Information 5. [file 41598_2022_13094_MOESM5_ESM.pdf]
