# Supplementary material for: Evaluation and comparison of antibiotic susceptibility profiles of Streptomyces spp. from clinical specimens revealed common and region-dependent resistance patterns
Source: Sci Rep. 2022 Jun 7;12:9353. doi: 10.1038/s41598-022-13094-4 (PMC9174267; doi:10.1038/s41598-022-13094-4)

Supplementary Figure S6. Penicillin. Results of correlation analysis of BM and DD methods followed by susceptibility testing of clinical isolates. A) Scattergram comparing the results of broth microdilution MICs (mg/L) and zone diameters (mm) for 49 *Streptomyces* strains. The lines represent the proposed ZD interpretive criteria. **B)** The graph depicts zone diameters distribution for 84 clinical *Streptomyces* strains, doted lines represents proposed zone diameter breakpoints (R - resistant category, S - susceptible category).

Note: Resistant isolates were predominantly available for the penicillin susceptibility tests, and only the soil isolate BCCO 10_1656 was considered susceptible (MIC = 0.12 mg/L, zone size 50 mm). Therefore, error rates were not calculated and ZD breakpoints were set as maximum/ minimum ZD values measured for the resistant/ susceptible MIC value.

A.

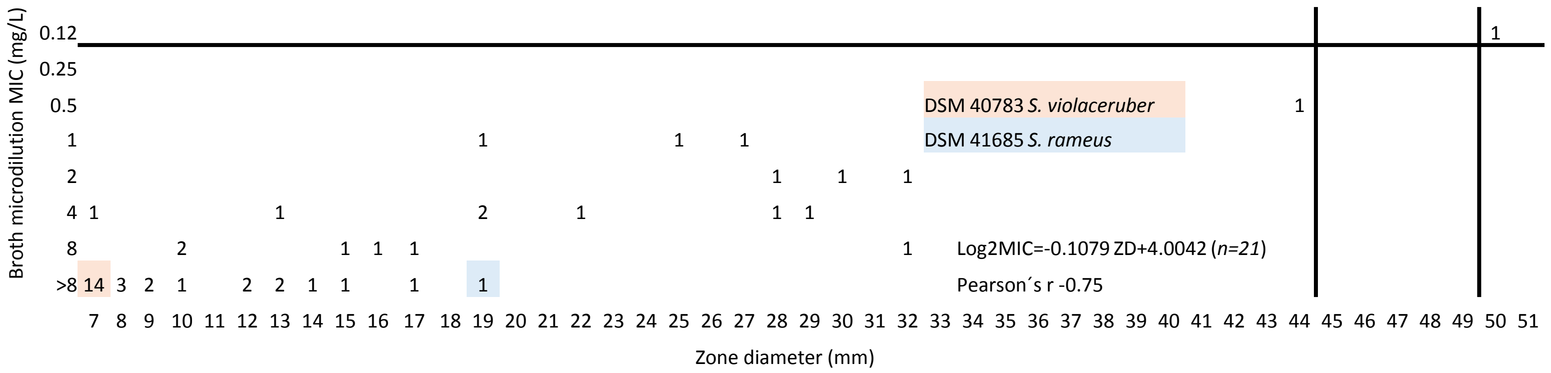

B.

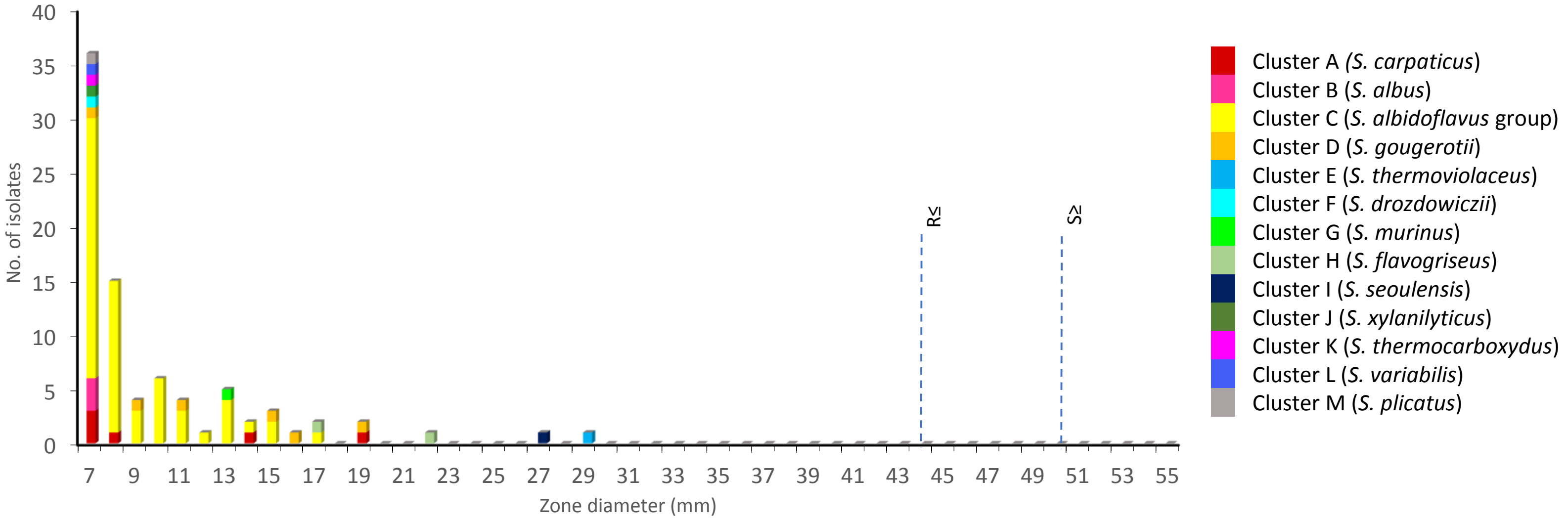

Supplement: Supplementary file 6 — Supplementary Information 6. [file 41598_2022_13094_MOESM6_ESM.pdf]
