# Supplementary material for: Evaluation and comparison of antibiotic susceptibility profiles of Streptomyces spp. from clinical specimens revealed common and region-dependent resistance patterns
Source: Sci Rep. 2022 Jun 7;12:9353. doi: 10.1038/s41598-022-13094-4 (PMC9174267; doi:10.1038/s41598-022-13094-4)

Supplementary Figure S7. Results of correlation analysis of BM and DD methods followed by susceptibility testing of clinical isolates. Vancomycin (A-B), gentamycin (C-D) and amikacin (E-F). Left panel: Scattergrams comparing the results of broth microdilution MICs (mg/L) and zone diameters (mm) for 49 *Streptomyces* strains. The lines represent the proposed ZD interpretative criteria. **Right panel:** The graph depicts zone diameters distribution for 84 clinical *Streptomyces* strains, doted lines represents proposed zone diameter breakpoint (S - susceptible category) and CO_{WT} value.

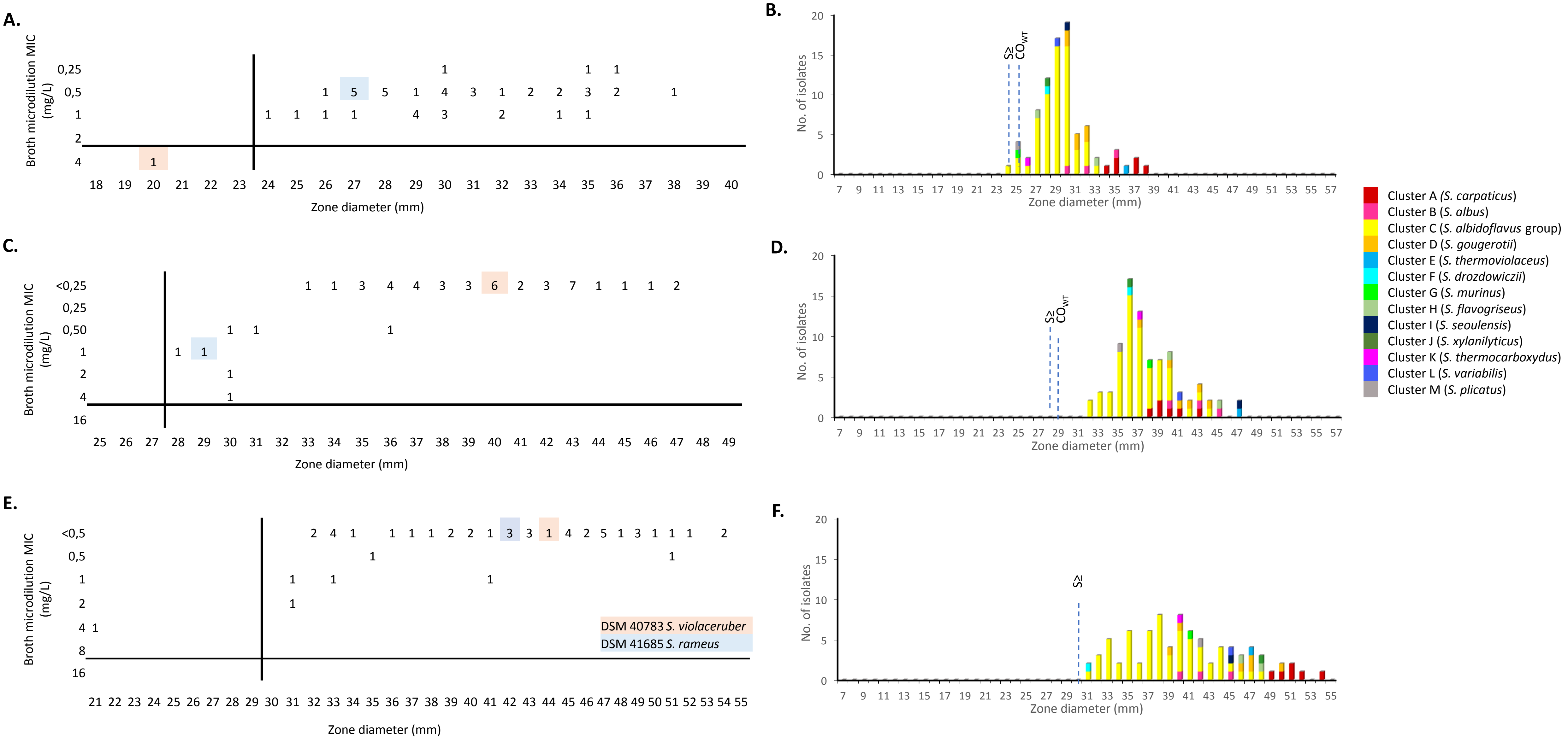

Supplement: Supplementary file 7 — Supplementary Information 7. [file 41598_2022_13094_MOESM7_ESM.pdf]
