# Supplementary material for: Evaluation and comparison of antibiotic susceptibility profiles of Streptomyces spp. from clinical specimens revealed common and region-dependent resistance patterns
Source: Sci Rep. 2022 Jun 7;12:9353. doi: 10.1038/s41598-022-13094-4 (PMC9174267; doi:10.1038/s41598-022-13094-4)

Supplementary Figure S8. Zone diameters distribution among the clusters, where correlations of MIC and ZD were not performed, however the S or R breakpoints can be proposed. Cefazolin (A), ceftriaxone (B), clarithromycin (C). The graphs depict zone diameters distribution for 84 clinical *Streptomyces* strains, doted lines represents proposed tentative zone diameter breakpoints (S - susceptible category, R – resistant category) and CO_{WT} value.

i) For both **cephalosporins (A, B)** the CO_{WT} of the dominant cluster C is very low indicating the presence of a resistant population. Since the ZD distributions for both antibiotics are very similar, the same tentative breakpoints corresponding to the maximum ZD of clusters C and D and the zone diameter distribution of the other strains were set.

ii) For **clarithromycin**, the ZD values of the dominant cluster C are indicating that the *S. albidoflavus* cluster is most likely sensitive. The other strains tested had similar or higher ZD values, with the exception of clusters A and B, where the strains seem to be more resistant or intermediate susceptible. We therefore used 26 mm as the breakpoint for the „S" category, R ≤ 21 mm and I = 22-25 mm.

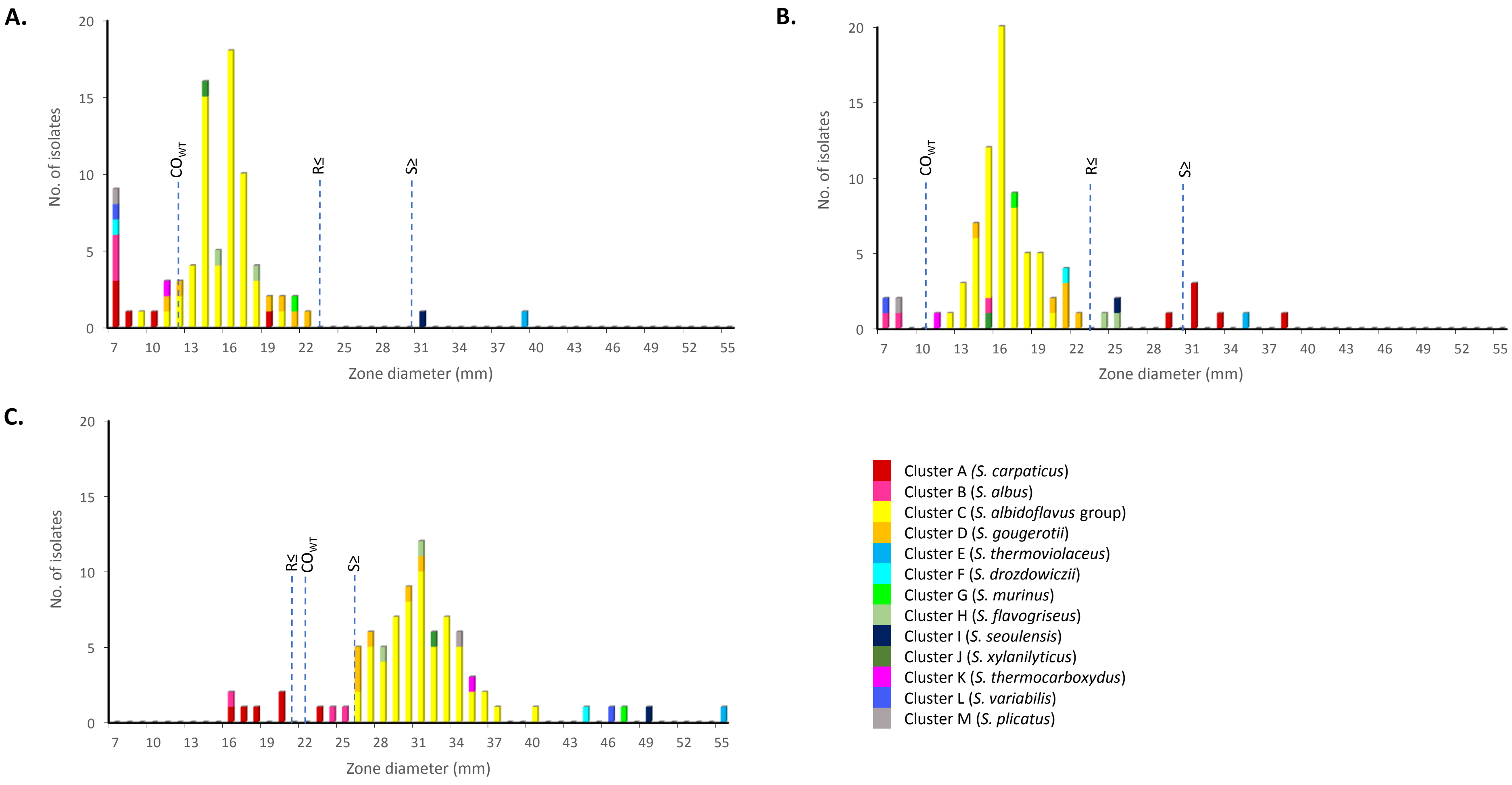

Supplement: Supplementary file 8 — Supplementary Information 8. [file 41598_2022_13094_MOESM8_ESM.pdf]
