# Supplementary material for: Evaluation and comparison of antibiotic susceptibility profiles of Streptomyces spp. from clinical specimens revealed common and region-dependent resistance patterns
Source: Sci Rep. 2022 Jun 7;12:9353. doi: 10.1038/s41598-022-13094-4 (PMC9174267; doi:10.1038/s41598-022-13094-4)

Supplementary Figure S9. Zone diameters distribution among the clusters, where correlation of MIC and ZD were not performed, however the S or R breakpoints can be proposed. Doxycycline (A), minocycline (B), amoxicillin (C) and amoxicillin-clavulanic acid (D). The graphs depict zone diameters distribution for 84 clinical *Streptomyces* strains, doted lines represents proposed tentative zone diameter breakpoints (S - susceptible category, R – resistant category) and CO_{WT} value.

i) Although the MIC breakpoints for **minocycline** and **doxycycline** are listed in the M24 guideline (CLSI), the broth microdilution method was not performed because these drugs were not included in commercial kits for AST. The zone diameter distributions of doxycycline and minocycline are very similar to those of tetracycline (CO_{WT} = 34 mm), so we used identical susceptibility breakpoints.

ii) To predict **amoxicillin** activity, CLSI and EUCAST recommend following the ampicillin susceptibility test in some cases (*Enterococcus sp., Haemophilus influenzae* by CLSI). In addition, the MIC breakpoints available in the guidelines are in some cases the same for both antibiotics (for enterococci and some streptococci, anaerobes and unrelated species). The ZD distribution of cluster C isolates for ampicillin and amoxicillin indicated the same susceptibility activity, although the disk content was different in our study (AMP 10 µg versus AMX 25 µg). In view of this, we derived the interpretive criteria for amoxicillin by adjusting the interpretive criteria for ampicillin to the difference between average ZD values of ampicillin and amoxicillin for 59 strains in cluster C (by 6 mm). **Amoxicillin-clavulanic acid** interpretive criteria were adapted from the amoxicillin breakpoints: strains whose growth was not affected by clavulanic acid have defined susceptibility breakpoints. These were the strains from cluster A and TR1318 from cluster H, where the decrease in ZD values was most likely due to lower content of amoxicillin in the disks and therefore we can assume that their growth was not affected by clavulanic acid supplementation.

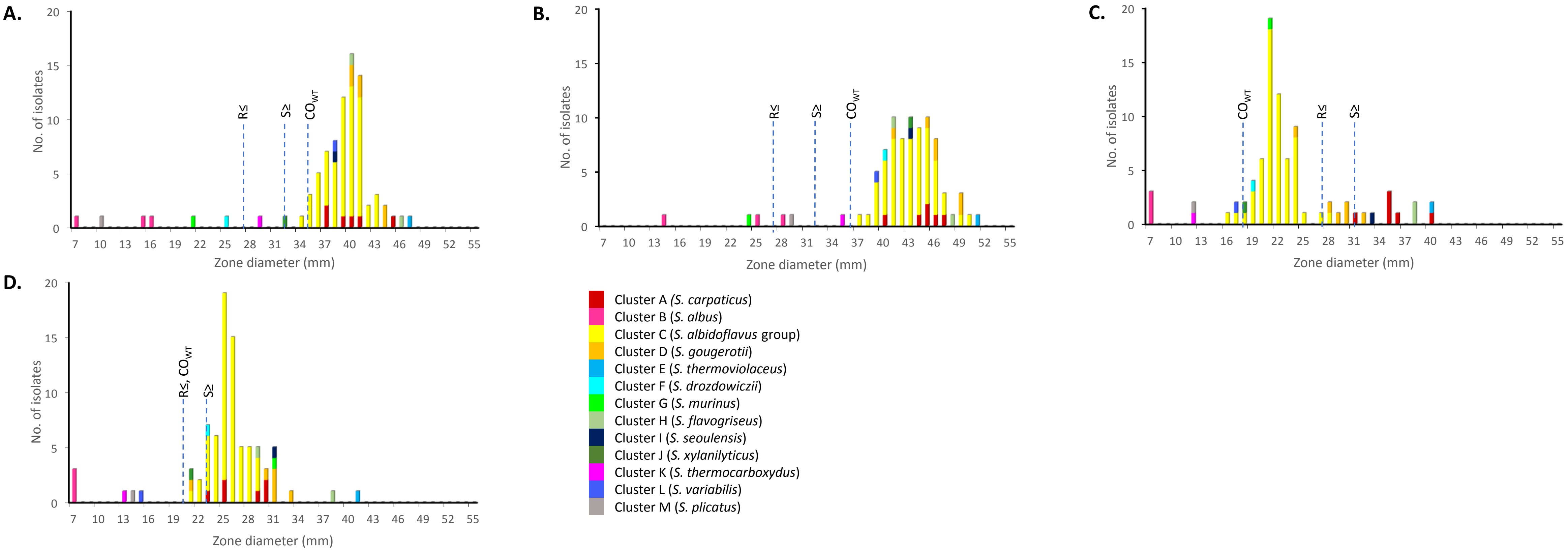

Supplement: Supplementary file 9 — Supplementary Information 9. [file 41598_2022_13094_MOESM9_ESM.pdf]
