# Supplementary material for: Evaluation and comparison of antibiotic susceptibility profiles of Streptomyces spp. from clinical specimens revealed common and region-dependent resistance patterns
Source: Sci Rep. 2022 Jun 7;12:9353. doi: 10.1038/s41598-022-13094-4 (PMC9174267; doi:10.1038/s41598-022-13094-4)

Supplementary Figure S10. Zone diameters distribution among the clusters, where correlation of MIC and ZD were not performed, S or R breakpoints are proposed only arbitrarily. Streptomycin (A), ofloxacin (B) and rifampicin (C). The graphs depict zone diameters distribution for 84 clinical *Streptomyces* strains, doted lines represents arbitrary proposed zone diameter breakpoints (S - susceptible category, R – resistant category) and CO_{WT} value.

i) The ZD values of most species tested ranged between 20-27 mm in the case of **streptomycin** and **ofloxacin**, which is usually the range of doubt. There are no obvious cases of susceptibility or resistance in the dominant cluster C.

ii) In the case of **rifampicin**, the ZD values of most species tested ranged between 24-45 mm, indicating susceptibility. However, due to the wide range of ZD values with no a significant susceptibility peak within cluster C, the data set has an abnormal SD for calculating the CO_{WT} value, so the difference between the wild type population and the population with acquired resistance mechanisms could not be determined. Variability in ZD values within the same phylotype was evident for other species as well (clusters A and B).

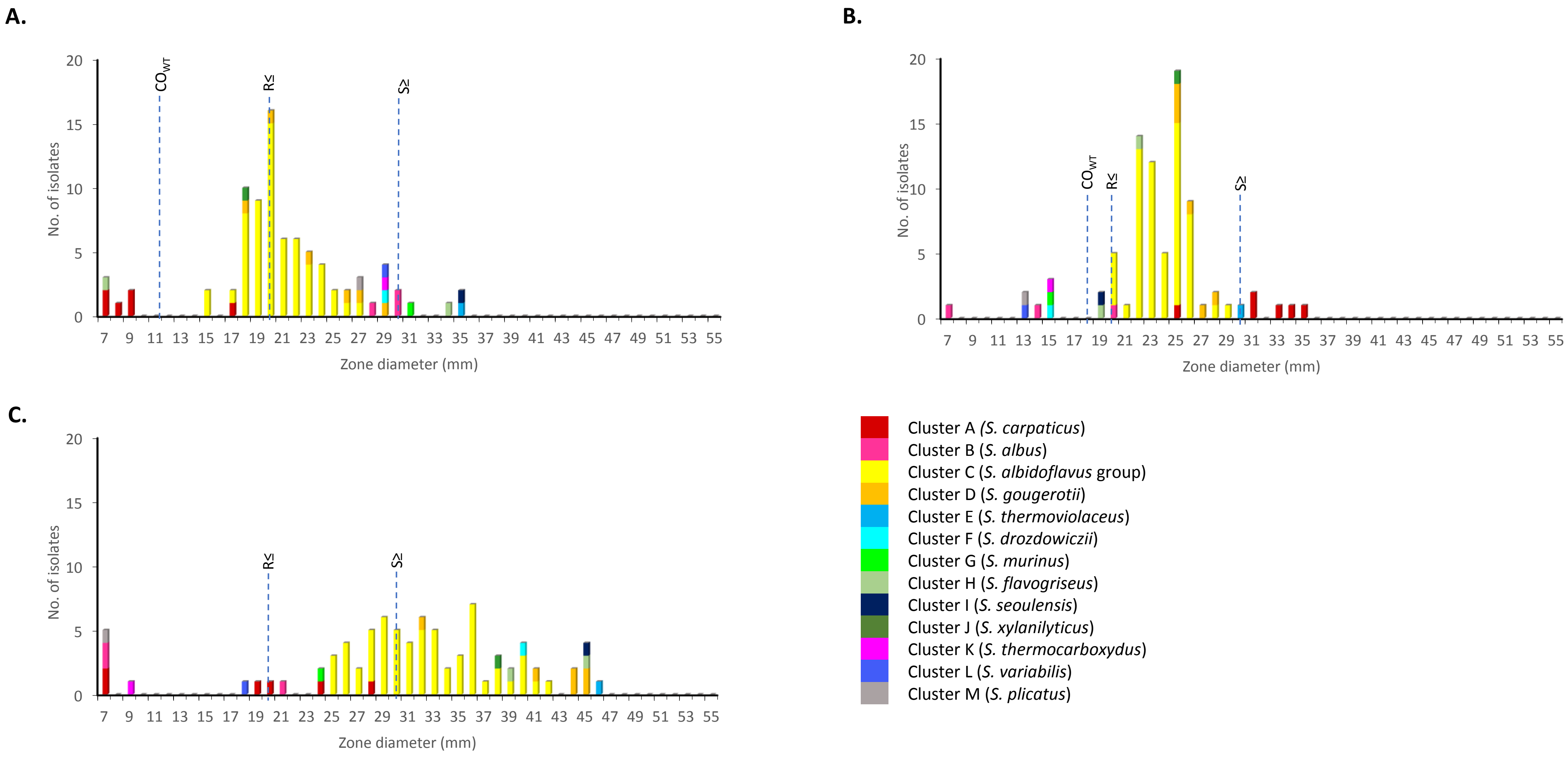

Supplement: Supplementary file 10 — Supplementary Information 10. [file 41598_2022_13094_MOESM10_ESM.pdf]
