# Supplementary material for: Evaluation and comparison of antibiotic susceptibility profiles of Streptomyces spp. from clinical specimens revealed common and region-dependent resistance patterns
Source: Sci Rep. 2022 Jun 7;12:9353. doi: 10.1038/s41598-022-13094-4 (PMC9174267; doi:10.1038/s41598-022-13094-4)

Supplementary Figure S11. Multi-drug resistance evaluation. Distribution of phenotypes resistant to 1-12 antibiotics among studied *Streptomyces* strains (20 drugs evaluated in total).

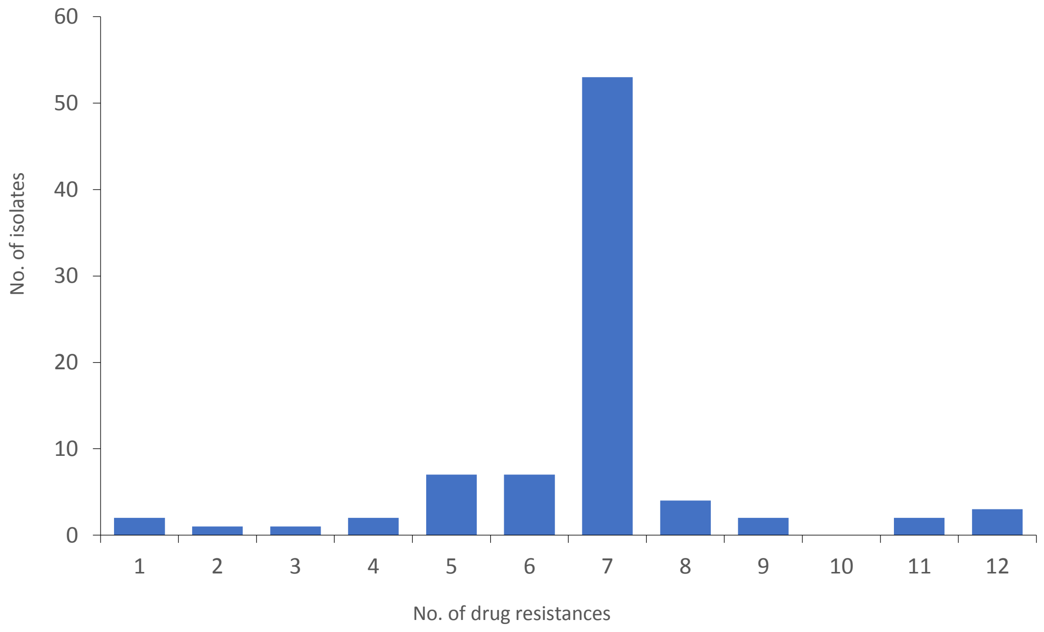

Supplement: Supplementary file 11 — Supplementary Information 11. [file 41598_2022_13094_MOESM11_ESM.pdf]
