# Supplementary material for: Evaluation and comparison of antibiotic susceptibility profiles of Streptomyces spp. from clinical specimens revealed common and region-dependent resistance patterns
Source: Sci Rep. 2022 Jun 7;12:9353. doi: 10.1038/s41598-022-13094-4 (PMC9174267; doi:10.1038/s41598-022-13094-4)
Supplement: Supplementary file 15 — Supplementary Information 15. [file 41598_2022_13094_MOESM15_ESM.docx]

**Supplementary Text S1**

**Evaluation and comparison of antibiotic susceptibility profiles of *Streptomyces* spp. from clinical specimens revealed common and region-dependent resistance patterns**

Kotrbová, L.^1^, Lara, A.C.^1^, Corretto, E.^1§^, Scharfen, J.^2^, Ulmann, V.^3^, Petříčková, K.^4^, Chroňáková, A.^1^*

^1^Institute of Soil Biology, Biology Centre Academy of Sciences of the Czech Republic, České Budějovice, Czechia

^2^National Laboratory for Pathogenic Actinomycetes, Trutnov Regional Hospital, Trutnov, Czechia

^3^Public Health Institute Ostrava, Ostrava, Czechia

^4^Institute of Immunology and Microbiology, 1st Faculty of Medicine, Charles University, Prague, Czechia

^§^Current address: Faculty of Science and Technology, Free University of Bozen-Bolzano, Bozen-Bolzano, Italy

Corresponding author:

Alica Chroňáková

Biology Centre CAS, v. v. i., Institute of Soil Biology

370 05 České Budějovice, Czech Republic

Phone: +420-387 775 770, Fax: +420-385 310 133, E-mail: alica.chronakova@upb.cas.cz

Running title: **ATB susceptibility of clinical *Streptomyces* spp.**

**Materials and methods**

**Bacterial isolates acquisition and identification**

All clinical strains (84) of *Streptomyces* spp. were isolated during routine diagnostics in mycobacteriology laboratories and were mainly isolated from patients with respiratory diseases (50 isolates), but 16 patient-derived isolates did not have a defined disease. A total of 75 strains were isolated from decontaminated sputum (DCSP) or bronchoalveolar lavage fluid (BAL) and for 6 isolates the tissue of origin was not defined. Clinical material from the respiratory tract was processed by standard homogenization and decontamination. Clinical strains were isolated on Löwenstein-Jensen egg media and in liquid media and cultured at 37 °C, followed by microscopic and macroscopic evaluation. DNA extraction and identification of all isolates was performed according to^1^, i.e. based on the nucleotide similarity of the 16S rRNA gene. The following modification was made: the sequences were compared with the GenBank sequences of type strains using the Basic Local Alignment Search Tool^2^ to identify the most closely related species. Species were then assigned to clusters based on a phylogenetic reconstruction performed using Geneious (v 8.1.6, http://www.geneious.com, neighbour joining, Tamura-Nei genetic distance model, 1000 replicates).

**Antibiotic susceptibility testing (AST)**

The two most common methods for AST are broth microdilution (BM) with interpretative criteria (breakpoints) defined as minimum inhibitory concentration (MIC; mg/L or μg/mL) and the disk diffusion (DD) with interpretative criteria defined as zone diameter values (ZD; mm). These breakpoints are used to categorize an organism as susceptible (S), intermediate (I) or resistant (R)^3^. A microorganism is categorized as: **S** if there is a high probability of therapeutic success using a standard antibiotic dosing regimen. **I,** if the microorganism is susceptible to increased exposure to the antibiotic and there is a high probability of therapeutic success after adjusting the dosing regimen or by increasing the antibiotic concentration at the site of infection. The intermediate susceptibility category also indicates a “buffer zone” to prevent small, uncontrolled technical factors from causing major discrepancies in the interpretation^4,5^. The **R** category indicates that the probability of treatment failure is high even with increased exposure to the antibiotic. Since *Streptomyces* species are better adapted to solid/semi-solid growth media^6^, and the BM method is rather resource-intensive and time-consuming method, we chose DD method.

1. **Antimicrobials**

Representatives of the major antibiotic categories were selected: aminoglycosides (amikacin, gentamycin, streptomycin), beta-lactams (cefazolin, ceftriaxone, penicillin, amoxicillin, ampicillin, amoxicillin-clavulanic acid), glycopeptides (vancomycin, teicoplanin), quinolones (ciprofloxacin, ofloxacin), rifamycin (rifampicin), amphenicol (chloramphenicol), macrolides (erythromycin, clarithromycin), oxazolidinone (linezolid), sulphonamide + pyrimidine (trimethoprim-sulfamethoxazole), and tetracyclines (tetracycline, doxycycline, minocycline). They comprised both bactericidal and bacteriostatic agents. The antimicrobials used for ZD determination are listed in Table 2. The disk contents are included.

1. **Disk diffusion test**

Isolates were grown on ISP3 medium^7^ containing (per liter): Oatmeal Agar (Himedia, Mumbai, India), 20 g; FeSO_4_·7H_2_O, 0.001 g; MnCl_2_·4H_2_O, 0.001 g; ZnSO_4_·7H_2_O, 0.001 g. Isolates were incubated at 36 °C for 7 days. A heavy suspension of each strain was prepared by adding a loop full of biomass to 500 µl of sterile saline in a sterile microcentrifuge tube with glass beads; vigorous vortexing ensured the disruption of clumped mycelia. The heavy suspension was then decanted, and the supernatant was used to prepare a 0.5 McFarland suspension (densitometer DEN-1, Biosan, Rīga, Latvia). The suspension (200 µL) was spread on Mueller-Hinton agar 90-mm plates (Dulab, Dubné, Czech Republic). Commercial antibiotic disks (Bio-Rad, Hercules, CA, USA) were applied with a disk dispenser (Bio-Rad), using no more than 3 disks per plate. Plates were incubated at 36 °C. Zones of inhibition were examined with the naked eye against a dark background, with the plate held 30 cm from the eye. Zones were measured after 24 or 48 hours, depending on the growth rate of the strains. In the case of trimethoprim-sulfamethoxazole, slight growth within the zone was ignored as recommended in guidelines for broth microdilution method^8^. All strains were tested in duplicate.

1. **Clinical breakpoints setting.**

To the best of our knowledge, there are no available breakpoints for the evaluation of AST results of *Streptomyces* spp. Therefore, it is necessary to choose susceptibility breakpoints from those recommended for the most related organisms. The Clinical and Laboratory Standard Institute (CLSI) recommends following the M24 guidelines^8^ for AST of *Streptomyces*, as these are the guidelines recommended for AST of *Nocardia* spp., even though they can be tentatively applied to all aerobic actinomycete groups of microorganisms, including *Streptomyces*. For antibiotics not included in the M24 guidelines, the MIC breakpoints for *Corynebacterium* spp. can be followed^9,10^, as it is a taxa related to *Streptomyces*. However, one of the important shortcomings of this approach is that only the broth microdilution methodology is suitable for *Streptomyces* AST [the European Committee for Antimicrobial Susceptibility Testing (EUCAST) also provides a ZD breakpoints, but the method is performed in 5 % CO_2_ atmosphere, which makes it inapplicable for *Streptomycetes* spp.] and MIC breakpoints are available only for a limited number of antibiotics. Therefore, we performed the BM method to set interpretive breakpoints by correlating the ZD values with BM MICs, as this was proved to be the most accurate method for setting of DD breakpoints. The correlation was performed only for the antibiotic of interest that was available in a commercial BM MIC kit. For the remaining antibiotics, the tentative breakpoints were set according to the distribution of the ZD data and the antibiotic class.

The MIC breakpoints for *Streptomyces* related taxa available in literature for antibiotics were used for correlations in our study and the concentration ranges of the BM method are listed in the Supplementary TablesS3. Discrepancies between the MIC breakpoint values given in different guidelines are obvious, even for the same bacterial taxon. Harmonizing breakpoints for common pathogenic bacteria has been precluded so far due to fundamental differences in the used databases, data interpretation, dosages used in different parts of the world, and public health policies^3^.

**Ampicillin**. As no MIC breakpoints are available in the guidelines, the tentative breakpoints from a previous study^11^ on antibiotic susceptibility of clinical *Nocardia* spp. were used and adapted for *Streptomyces* susceptibility evaluation. **Ciprofloxacin**. In our study, we primarily followed the MIC breakpoints for aerobic actinomycetes described in M24 guidelines^8^ when possible. However, the MIC breakpoints for *Corynebacterium* spp. reported by the European Committee for Antimicrobial Susceptibility Testing (EUCAST) are also available, as *Corynebacterium* spp. belong to the aerobic actinomycete group and are related to the *Streptomyces* genus. In the comparison, the “S” category of the EUCAST breakpoints for *Corynebacterium* spp. remains the same as the “S” category defined by the CLSI, immediately followed by the “R” category, whereas the “I” category is omitted in the EUCAST guidelines. **Chloramphenicol**. Because no MIC breakpoints for chloramphenicol are available in literature for any genus related to *Streptomyces*, we followed MIC breakpoints reported in a previous study of clinical *Corynebacterium* spp.^12^. **Erythromycin**. The MIC breakpoints proposed by the CLSI^8^ for clarithromycin were used to propose zone diameter breakpoints, as this drug represents the class of newer macrolides. Although erythromycin belongs to the first generation of macrolides, the breakpoints for the “S” category are the same for both antibiotics when used for *Corynebacterium* spp.^9,10^, and even for most of the genera stated in the EUCAST manual^10^. **Tetracycline**. There are three MIC breakpoints that can be used to determine ZD breakpoints: i) MIC breakpoints for *Corynebacterium* ssp. in the EUCAST guidelines, and ii) MIC breakpoints for *Corynebacterium* ssp. in the CLSI guidelines, which surprisingly differ by one MIC value in the „S” category and by two MIC values in the „R” category, and the iii) MIC breakpoints for aerobic actinomycetes from CLSI for minocycline and doxycycline, as they are representatives of the tetracycline class. When comparing the MIC breakpoints for tetracycline and minocycline (doxycycline) for other gram-positive bacteria, differences in guidelines, in the pharmacokinetics and pharmacodynamics of members of the tetracycline group are reported. Therefore, the breakpoints reported in the CLSI M24 guidelines were not followed. Based on the distribution of MIC and ZD values in our study and the fact that the strains originated from Central Europe, we followed EUCAST MIC breakpoints for *Corynebacterium* spp.^10^. **Penicillin**. Two acceptable MIC breakpoints are given in the CLSI and EUCAST guidelines for ZD breakpoint evaluation, both for *Corynebacterium* spp. The difference is in the „R” category: R ˃ 0.125 mg/L for EUCAST and R ≥ 4 mg/L for CLSI. The category „S” remains the same (S ≤ 0.12 mg/L). Since our study was performed in Central Europe, we used the more stringent breakpoints provided by EUCAST^10^.

1. **Minimum inhibitory concentration (MIC) determinations**

The broth microdilution method for aerobic *Actinomycetes* described by CLSI in the M24 manual^8^ was performed according to the guidelines. A total of 29 clinical isolates were tested with the BM method. The number of strains of a given cluster destined for this analysis was selected proportionately. To increase the robustness of the correlation analysis, 18 additional soil isolates of *Streptomyces* spp. (also deposited in the Culture Collection of Soil Actinomycetes, www.actinomycetes.bcco.cz) and the type strain *S. rameus* DSM 41685 and *S. violaceoruber* DSM 40783 were included in the study. The environmental isolates followed the same protocol as the clinical isolates (DD method included), except for the incubation temperature, which was lowered to 28°C.

Isolates were cultivated at 36 °C (28°C for environmental isolates) in ambient air for 5-7 days prior to testing on Tryptic Soya Agar (Oxoid, Hampshire, England) plates or on Mueller-Hinton agar supplemented with 5 % of defibrinated sheep blood (Dulab, Dubné, Czech Republic). A total of 65 µL of the 0.5 McFarland suspension (preparation as described above) was mixed with 13 mL of Cation-adjusted Mueller-Hinton II Broth (CAMHB, Erba Lachema, Brno, Czech Republic) to obtain approximately 1.0 x 10^5^ to 5.0 x 10^5^ CFU/mL. The commercial antibiotic susceptibility testing kits MIKROLATEST MIC® (Erba Lachema, Brno, Czech Republic) with dried antibiotics were used to determine the minimum inhibitory concentrations (MIC). Each well in the microplate containing the antibiotic was covered with 100 µL of the MIC suspension. The last drop of the suspension was cultivated on Tryptic Soya Agar as a purity check. The inoculated panels were covered with an adhesive seal and incubated at 36°C in ambient air for 3 days. The assay was examined after 24, 48 and 72 hours to evaluate the growth. Results were read after 72 hours, as the growth of all strains was optimal at this time. The MIC endpoint (mg/L) was the lowest concentration that inhibited visible growth, except for the trimethoprim-sulfamethoxazole endpoint. The well with approximately 80 % growth inhibition compared to growth in the positive control well was determined to be the trimethoprim-sulfamethoxazole endpoint. Endpoints were evaluated visually by naked eye against a dark background. All strains were tested in duplicate and if inconsistency of MIC endpoint occurred between duplicates of the same drug, only the higher endpoint was reported. If a discrepancy of more than one MIC endpoint occurred between duplicates, the MIC determination was repeated. As recommended by MIKROLATEST MIC®, *Enterococcus faecalis* DSM 2570 and *Escherichia coli* DSM 1103 were used as quality controls. The MIC interpretive criteria listed in Table 2 were followed when evaluating the results of the DD susceptibility test.

**Data analysis**

The correlation between binary logarithms of the MIC endpoints values (mg/L) and average zone diameters (mm) was tested using the Pearson correlation coefficient. The accuracy limit was set at r > 0.75. Off-scale values were excluded. Zone diameter interpretive susceptibility criteria were derived from the scattergrams of MIC endpoints and the ZD values followed by the error rate-bounded method^4^. The discrepancy percentage between the correlated methods was calculated as very major error (VM; false-susceptible by disk diffusion), major error (M; false-resistant by disk diffusion) and minor error (m; one of the test results is intermediate and the other is susceptible or resistant). In addition to clinical breakpoints used to guide the therapy, the European Committee for Antimicrobial Susceptibility Testing (EUCAST) has defined the concept of epidemiological cut-off values, which are thresholds for detecting bacteria with resistance mechanisms and for monitoring the evolution of resistance among isolates of the same bacterial species. Epidemiological cut-off values are the MIC values of a drug that divides a given bacterial population into a wild type group and a group with acquired or mutational resistance to that drug. This value is one of the relevant factors in setting official breakpoints for antimicrobial agents^13^ and thus the breakpoints were determined by automatic calculation for zone distributions using the of Normalized Resistance Interpretation^14^ method. The automatic and manual excel programs were made available through courtesy of P. Smith, W. Finnegan, and G. Kronvall (http://www.bioscand.se/nri/). As recommended, the epidemiological cut-off values calculated by this software were referred to by the acronym CO_WT_, as the terms epidemiological cut-off values (ECOFF and ECV) should be reserved for those defined by EUCAST and CLSI, respectively. The NRI method was used with permission from the patent holder, Bioscand AB, TÄBY, Sweden (European patent No. 1383913, US Patent No. 7,465,559). The obtained frequencies of resistant phenotypes were compared with those reported in a recent study^13^ of clinical isolates from Spain. The concordance between resistance profiles for overlapping antibiotics was evaluated by Pearson´s chi-squared test (α = 0.01). Only antibiotics with available MIC-DD correlation were included in the comparison (CIP, ERY and SXT). The frequency of resistance to the tested antibiotics, except those with arbitrary breakpoints (SMN, RIF and OFX), was calculated to determine multidrug resistance patterns of the clinical isolates.

**(vi) Antibiograms review.** Since the studies from clinical branches are presumed to use a standardized methods for AST evaluation, we targeted only for antibiograms reported for clinical *Streptomyces*. If the MIC or ZD values were stated in study^15–19^, we evaluated the susceptibility profile using the breakpoints cited or derived in our study.

**References**

1. Petříčková, K. *et al.* Evolution of cyclizing 5-aminolevulinate synthases in the biosynthesis of actinomycete secondary metabolites: Outcomes for genetic screening techniques. *Front. Microbiol.* **6**, 1–15 (2015).

2. Altschul, S. F., Gish, W., Miller, W., Myers, E. W. & Lipman, D. J. Basic local alignment search tool. *J. Mol. Biol.* **215**, 403–410 (1990).

3. CLSI. Performance standards for antimicrobial susceptibility testing. 30th Ed. *CLSI guidlines* *M100* http://em100.edaptivedocs.net/GetDoc.aspx?doc=CLSI M100 ED31:2021&scope=user (2020).

4. CLSI. Development of in vitro susceptibility testing criteria and quality control parameters. 2nd Ed. *CLSI guidlines* *M23* (2001).

5. EUCAST. New definition of S, I and R from 2019. *www.eucast.org/newsiandr/* (2020).

6. Kieser, T., Bibb, M. J., Buttner, M., Chater, K. F. & Hopwood, D. Growth and preservation of *Streptomyces*. *Pract. Streptomyces Genet.* 43–62 (2000).

7. Shirling, E. B. & Gottlieb, D. Methods for characterization of *Streptomyces* species. *Int. J. Syst. Evol. Microbiol.* **16**, 313–340 (1966).

8. CLSI. Susceptibility testing of *Mycobacteria,* and other aerobic *Actinomycetes*. 2nd Ed. *CLSI guidlines* M24 (2011).

9. CLSI. Methods for antimicrobial dilution and disk susceptibility testing of infrequentlyisolated or fastidious bacteria. 3rd Ed. *Guidelines CLSI M45* (2015).

10. EUCAST. Testing Breakpoint tables for interpretation of MICs and zone diameters. *Https://Www.Eucast.Org/Ast_of_Bacteria/* 0–77 (2020).

11. Larruskain, J., Idigoras, P., Marimón, J. M. & Pérez-Trallero, E. Susceptibility of 186 *Nocardia* sp. isolates to 20 antimicrobial agents. *Antimicrob. Agents Chemother.* **55**, 2995–2998 (2011).

12. Dragomirescu, C. C. *et al.* Antimicrobial susceptibility testing for *Corynebacterium* species isolated from clinical samples in Romania. *Antibiotics* **9**, 1–9 (2020).

13. EUCAST. Harmonizing of breakpoints for existing antimicrobial agents. *EUCAST standard operation procedure* www.eucast.org (2019).

14. Kronvall, G. & Smith, P. Normalized resistance interpretation, the NRI method: Review of NRI disc test applications and guide to calculations. *Apmis* **124**, 1023–1030 (2016).

15. McNeil, M. M., Brown, J. M., Jarvis, W. R. & Ajello, L. Comparison of species ditribution and antimicrobial susceptibility of aerobic actinomycetes from clinical specimens. *Rev. Infect. Dis.* **12**, 778–783 (1990).

16. Nasher, M. A., Hay, R. J., Mahgoub, E. S. & Gumaa, S. A. In vitro studies of antibiotic sensitivities of *Streptomyces somaliensis* a cause of human actinomycetoma. *Trans. R. Soc. Trop. Med. Hyg.* **83**, 265–268 (1989).

17. Manteca, A. *et al.* A rare case of lung coinfection by *Streptomyces cinereoruber* and *Haemophilus influenzae* in a patient with severe chronic obstructive pulmonary disease: characterization at species level using molecular techniques. *Diagn. Microbiol. Infect. Dis.* **60**, 307–311 (2008).

18. Chaudhuri, B. N., Maiti, P. K. & Sil, J. Antibiotic sensitivity patterns of actinomycetes isolated from patients of actinomycetoma. *Indian J. Med. Res.* **105**, 162—166 (1997).

19. Ai, L. *et al.* Chronic suppurative otitis media due to *Streptomyces cacaoi*, the second case report in human infection. *BMC Infect. Dis.* **20**, 10–13 (2020).
